# Phosphorelay through the bifunctional phosphotransferase PhyT controls the general stress response in an alphaproteobacterium

**DOI:** 10.1371/journal.pgen.1007294

**Published:** 2018-04-13

**Authors:** Lisa Gottschlich, Miriam Bortfeld-Miller, Christoph Gäbelein, Sebastian Dintner, Julia A. Vorholt

**Affiliations:** Institute of Microbiology, ETH Zurich, Zurich, Switzerland; University of Geneva Medical School, SWITZERLAND

## Abstract

Two-component systems constitute phosphotransfer signaling pathways and enable adaptation to environmental changes, an essential feature for bacterial survival. The general stress response (GSR) in the plant-protecting alphaproteobacterium *Sphingomonas melonis* Fr1 involves a two-component system consisting of multiple stress-sensing histidine kinases (Paks) and the response regulator PhyR; PhyR in turn regulates the alternative sigma factor EcfG, which controls expression of the GSR regulon. While Paks had been shown to phosphorylate PhyR *in vitro*, it remained unclear if and under which conditions direct phosphorylation happens in the cell, as Paks also phosphorylate the single domain response regulator SdrG, an essential yet enigmatic component of the GSR signaling pathway. Here, we analyze the role of SdrG and investigate an alternative function of the membrane-bound PhyP (here re-designated PhyT), previously assumed to act as a PhyR phosphatase. *In vitro* assays show that PhyT transfers a phosphoryl group from SdrG to PhyR via phosphoryl transfer on a conserved His residue. This finding, as well as complementary GSR reporter assays, indicate the participation of SdrG and PhyT in a Pak-SdrG-PhyT-PhyR phosphorelay. Furthermore, we demonstrate complex formation between PhyT and PhyR. This finding is substantiated by PhyT-dependent membrane association of PhyR in unstressed cells, while the response regulator is released from the membrane upon stress induction. Our data support a model in which PhyT sequesters PhyR, thereby favoring Pak-dependent phosphorylation of SdrG. In addition, PhyT assumes the role of the SdrG-phosphotransferase to activate PhyR. Our results place SdrG into the GSR signaling cascade and uncover a dual role of PhyT in the GSR.

## Author summary

The general stress response (GSR) in alphaproteobacteria represents an essential feature for survival in stressful, constantly changing habitats. A variety of stresses are sensed by GSR-activating histidine kinases and lead to multiple stress resistance via the response regulator PhyR. Here, we describe the essential bifunctional regulator PhyT, which tunes GSR activation in the plant-protecting strain *Sphingomonas melonis*. It prevents lethal over activation of the GSR by impeding inappropriate phosphorylation of the response regulator PhyR via protein-protein interaction. In addition, PhyT acts as a phosphotransferase in a GSR activating phosphorelay. Our data suggest a model according to which histidine kinases are induced by environmental cues, which results in phosphorylation of the single domain response regulator SdrG. The phosphoryl group of SdrG is then transferred by the phosphotransferase PhyT to the master regulator of the general stress response PhyR. Histidine kinase derived PhyT-type regulators are found also in other alphaproteobacteria, implying that the identified regulatory strategy might be conserved.

## Introduction

Two-component regulatory pathways enable bacteria to react to changing environmental conditions. Classical two-component systems consist of a sensor histidine kinase and a response regulator; the histidine kinase autophosphorylates upon sensing appropriate environmental signals and subsequently transfers the phosphoryl group to the response regulator, which in turn triggers an adaptation response [1–4]. Multicomponent phosphorelays represent more complex two-component systems involving either a so-called hybrid histidine kinase or, alternatively, a single domain response regulator (SDRR) which can be phosphorylated by multiple histidine kinases. Either way, a phosphotransferase subsequently transfers the phosphoryl group to an output response regulator. Therefore, by increasing the number of checkpoints in a phosphorylation pathway, phosphorelays allow for more precise regulation, e.g. [1–8].

The general stress response (GSR) is pivotal for alphaproteobacteria for environmental adaption and host microbe interactions [9, 10]. Notably, it connects a two-component system to alternative sigma-factor regulation [11, 12]. The GSR can be induced by a variety of different stresses and results in multiple stress resistance [9, 10]. Studied systems involve for example plant-associated bacteria [13] such as *Sphingomonas melonis* Fr1 [14], *Methylobacterium extorquens* [15], *Bradyrhizobium diazoefficiens* [16], *Sinorhizobium meliloti* [17], intracellular pathogens like *Brucella abortus* [11], or free-living species like *Caulobacter crescentus* [18]. The anti-sigma factor antagonist PhyR is phosphorylated under stressful conditions [14, 19] and acts via a mechanism termed "sigma factor mimicry" [20, 21]. A phosphorylation-induced conformational change of PhyR results in the release of its sigma-factor like domain that subsequently binds to the anti-sigma factor NepR. Thereby, NepR liberates the alternative sigma-factor EcfG, which can then bind to RNA polymerase and re-direct transcription towards the GSR regulon [20, 21]. In the plant-protecting *S*. *melonis* Fr1 [22, 23], seven cytoplasmic histidine kinases (Paks) are involved in the GSR [19]. They belong to the HWE/HisKA_2 family [19, 24], encoding the HRxxN motif in their DHp domain. Paks integrate multiple stress signals [19]. Individual stresses are sensed by one or more Paks, and some of the Paks are able to sense more than one stress. *In vitro*, the Paks do not only phosphorylate PhyR (in the presence of NepR), but also the SDRR SdrG [19].

The dual specificity of the Paks for PhyR and SdrG is supported at the structural level; in fact, the receiver domain of PhyR is the best structural homolog of SdrG [25]. Like Sma0144 from *S*. *meliloti* [26], SdrG belongs to the FAT GUY family of response regulators [25]. The SDRR is a key regulator of the GSR in *S*. *melonis* Fr1 [19] and *M*. *extorquens* [27]; however, its function can be bypassed by overexpression of either PhyR or the Paks [19]. The exact role of SdrG in GSR remains enigmatic. Potential mechanisms of SdrG activity might involve protein-protein interaction, comparable e.g. to CheY from *Escherichia coli*, which is involved in chemotaxis regulation [28, 29] or to DivK from *C*. *crescentus*, in which the regulator is involved in cell cycle control [30]. Alternatively, it is conceivable that SdrG participates in phosphotransfer, e.g. as the SDRR Spo0F, which is involved in sporulation initiation in *Bacillus subtilis* [5]; however, no phosphotransferase corresponding to Spo0B of *B*. *subtilis* has been identified for SdrG.

While in *S*. *melonis* Fr1 the genes encoding SdrG and the Paks are scattered throughout the genome, PhyP, an additional regulator of the GSR, is encoded at the *phyR* locus. The gene encodes a predicted membrane-anchored histidine kinase with a putative periplasmic sensor domain [14]. Closer inspection revealed that PhyP contains the HRxxN motif in its DHp domain, but harbors a degenerated ATPase domain [9, 14]. Due to its observed ability to disrupt the PhyR/NepR complex *in vitro* in a phosphorylatable His-341-dependent fashion, it has been proposed that PhyP acts as a PhyR phosphatase [14]. A *phyP* knockout was only obtained in GSR impaired strains, that is, in *phyR*, *ecfG*, or multiple *pak* knockout mutants, confirming PhyP as an essential negative regulator of the GSR in *S*. *melonis* Fr1 [14, 19].

The identification of PhyP-type proteins in other alphaproteobacteria, e.g. Bab1_1673 in *B*. *abortus* [11], RpaI_4707 in *Rhodopseudomonas palustris* TIE-1 [31], RsiC in *S*. *meliloti* [17], and LovK in *C*. *crescentus* [18] suggests a conserved key role of the protein in the GSR.

Here, we investigate the role of PhyP (re-designated PhyT in this study, see below) in *S*. *melonis* Fr1 and identify a direct molecular link to SdrG. Our results support a GSR regulatory model in which PhyT acts as a phosphotransferase mediating phosphotransfer between Pak-phosphorylated SdrG and PhyR. Furthermore, our results suggest that PhyT simultaneously prevents lethal over activation of the GSR by inhibiting direct PhyR phosphorylation by Paks via complex formation. The dual function of PhyT explains its essentiality and assigns a key function to SdrG.

## Results

### Identification of an SdrG-phosphotransferase as part of a phosphorelay to activate the GSR

In *S*. *melonis* Fr1, the *phyR* locus encodes the membrane-anchored protein PhyP, previously proposed to be a PhyR phosphatase, which is essential in strains with a functional GSR [14]. Here, we studied the function of this central regulator further. First, we set out to test *in vitro* PhyR dephosphorylation by PhyP using PhyR ^32^P-labeled at the Asp-194 residue [20, 21], which was generated in presence of NepR using one of the PhyR-activating kinases (here PakF) [19]. For dephosphorylation, we used *E*. *coli* membrane particles containing heterologously produced PhyP or the PhyP (H341A) derivative as a control [14]. However, no phosphatase activity was detected (S1 Fig).

**Fig 1:**
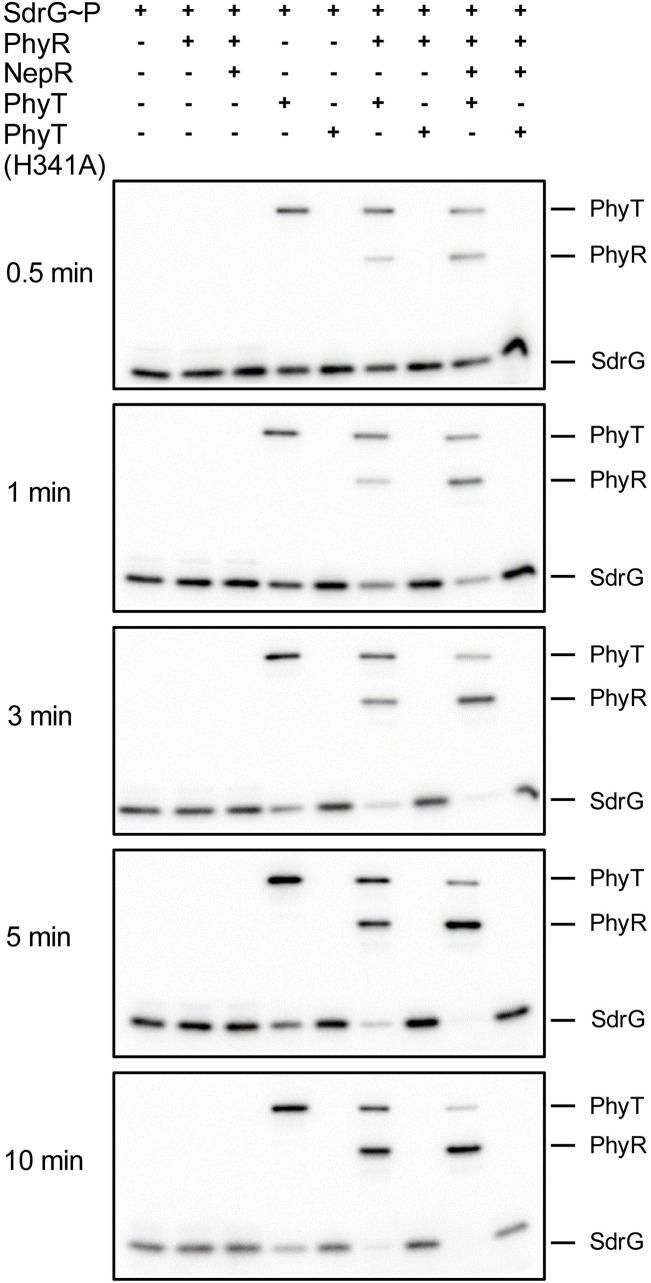
PhyT (formerly PhyP) transfers phosphoryl groups from SdrG~P to PhyR. *In vitro* phosphotransfer from SdrG~P to PhyT and further to PhyR in absence and presence of NepR over time. SdrG (10 μM) was phosphorylated using PakF (autophosphorylated with [γ-32P] ATP) on Ni-NTA columns. PhyR (5 μM), NepR (7.5 μM) and *E*. *coli* membrane particles (5 mg membrane fraction/mL) harboring either wild-type PhyT or the PhyT (H341A) derivative as a control were added as indicated. For confirmation of comparable amounts of PhyT and PhyT (H341A), Western blot analysis was conducted (S2A Fig).

We tested an alternative hypothesis regarding the function of PhyP that integrates the central, but not yet understood, role of the positive GSR regulator SdrG [19]. We speculated that PhyP could participate in the GSR-activating phosphotransfer and would assume the role of a PhyR-activating phosphotransferase rather than that of a PhyR phosphatase. According to our working model, Paks act as the primary phosphoryl group sources for SdrG [19]. The phosphoryl group could then be shuttled to PhyP and subsequently transferred to PhyR. To test such a putative phosphorelay, we performed time course *in vitro* phosphotransfer assays. To guarantee SdrG~P as the sole phosphodonor in the reaction mixture, we used an on-column phosphorylation approach, in which SdrG was phosphorylated by Ni-NTA-bound ^32^P-labeled PakF. The addition of SdrG~P to *E*. *coli* membrane particles harboring heterologously produced PhyP resulted in increasing PhyP phosphorylation and a concomitant decrease of SdrG~P (Fig 1). When SdrG~P was added to a mixture of PhyR and PhyP, SdrG~P dephosphorylation could be observed over time, while the level of PhyR~P increased. This transfer was enhanced in presence of the anti-sigma factor NepR, which is required for efficient direct phosphotransfer from Paks to PhyR *in vitro* [19]. No phosphotransfer from SdrG to PhyR was detectable in the absence of PhyP. Moreover, no decrease in SdrG~P and thus no phosphotransfer was observed for the inactive PhyP (H341A) derivative (Fig 1). Our *in vitro* data thus indicate that PhyP acts as a phosphotransferase that shuttles phosphoryl groups from SdrG~P to PhyR. Therefore, we have chosen to rename PhyP as PhyT and refer to it accordingly from now on.

**Fig 2:**
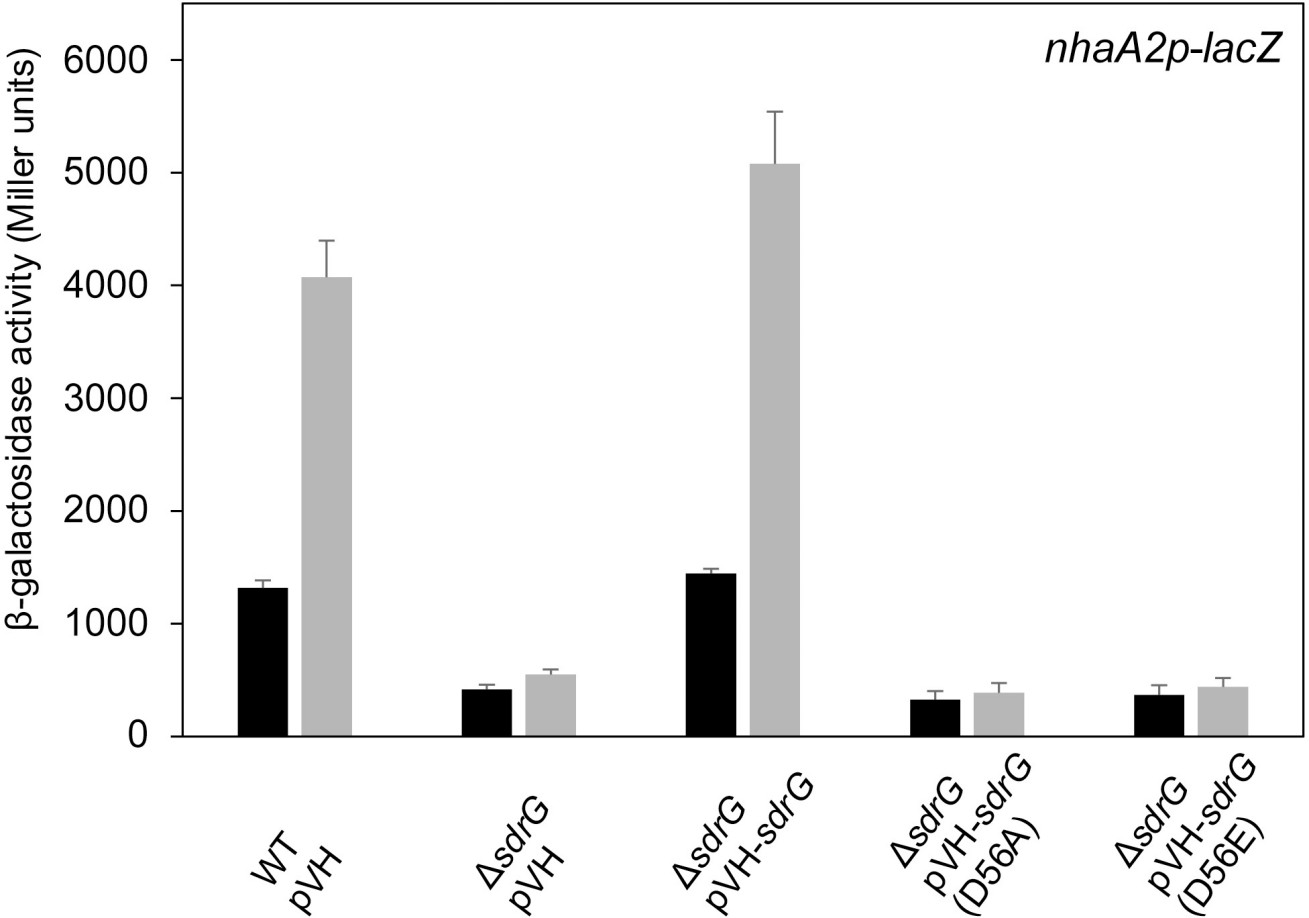
Phosphorylatable SdrG is essential for its positive regulatory role. β-galactosidase activity of the EcfG-dependent *nhaA2p-lacZ* fusion in indicated *S*. *melonis* Fr1 mutant backgrounds upon overnight overexpression of *sdrG* and variants from the vanillate-inducible pVH vector with 250 μM vanillate. pVH was used as empty-vector control. Black bars and gray bars represent β-galactosidase activity pre- and 1 h post-induction with the chemical stress mixture (1% ethanol, 80 mM NaCl and 50 μM TBHP), respectively. Values are given as mean ±SD of three independent experiments.

Next, we aimed to confirm that SdrG takes part in PhyR phosphorylation in presence of PhyT *in vivo* by testing whether the phosphorylatable Asp residue of SdrG is required to fulfill its role as a positive GSR regulator. Therefore, we performed EcfG-dependent β-galactosidase assays and compared *sdrG* knockout strains producing either wild-type SdrG or the SdrG (D56E) derivative encoding a Glu substitution of the phosphorylatable Asp-56 [25], a substitution that renders many response regulators active by mimicking the phosphorylated state [32–34]. Only wild-type SdrG rescued the impaired GSR observed for the *sdrG* knockout mutant. The SdrG (D56A) derivative was used as a negative control (Fig 2). This observation confirms previous work that demonstrated the inability of the SdrG (D56E) derivative to complement the salt-sensitive phenotype of an *sdrG* knockout strain [19] and suggests that SdrG phosphorylation is essential for its positive regulatory function in GSR.

Taken together, our data indicate that PhyT acts as a phosphotransferase in the Pak-SdrG-PhyT-PhyR phosphorelay of the GSR activating pathway in *S*. *melonis* Fr1.

### Role of the Pak-SdrG-PhyT-PhyR phosphorelay for stress-induced GSR activation *in vivo*

**Fig 3:**
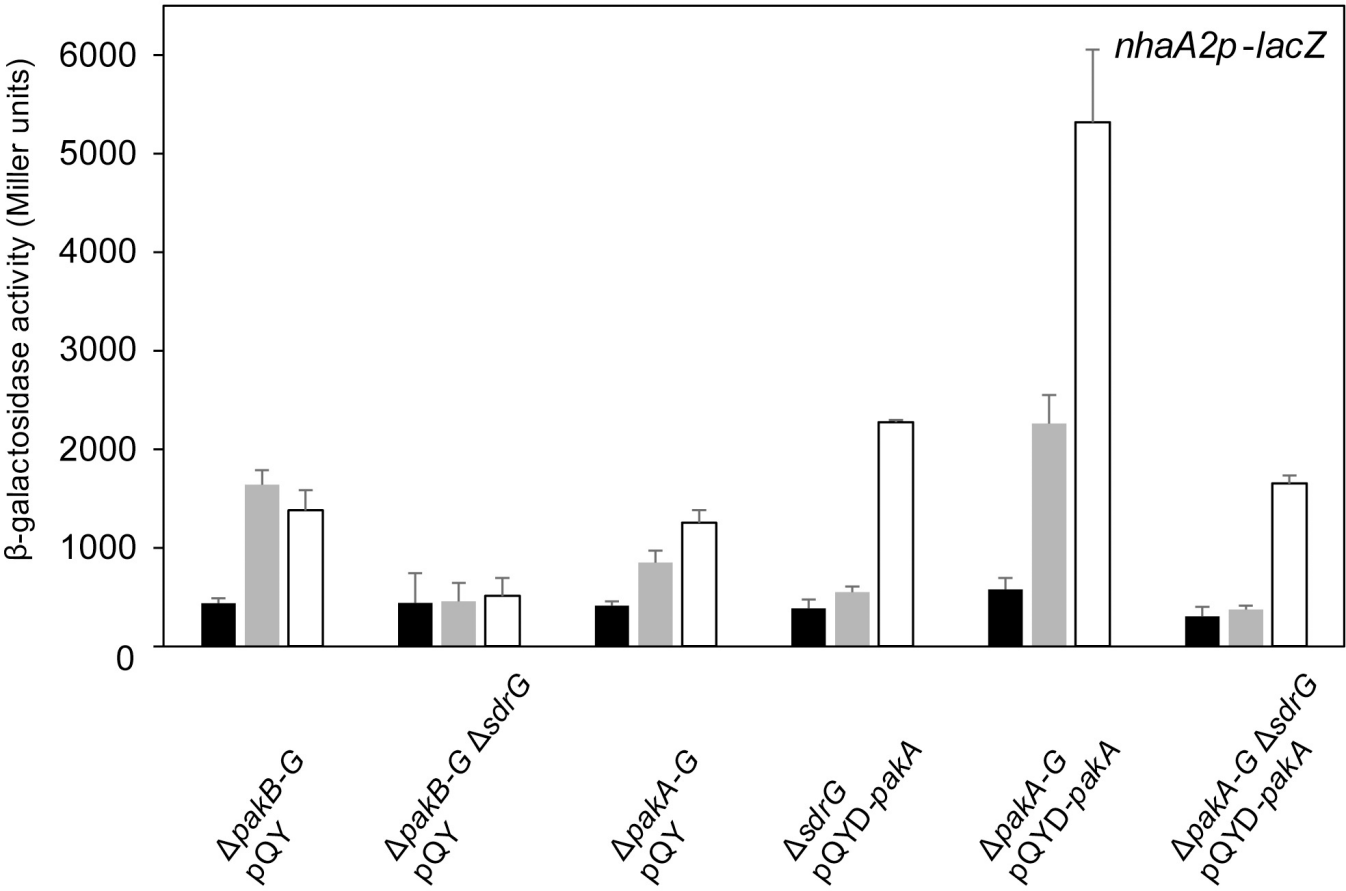
SdrG is important for stress induction of GSR. β-galactosidase activity of the EcfG-dependent *nhaA2p-lacZ* fusion in indicated *S*. *melonis* Fr1 mutant backgrounds upon overexpression of *pakA* or *sYFP2* from the cumate-inducible pQY vector. Black bars and gray bars represent β-galactosidase activity pre- and 1 h post-induction with the chemical stress mixture (1% ethanol, 80 mM NaCl and 50 μM TBHP), respectively; white bars represent β-galactosidase activity 3 h post-induction with chemical stress mixture and 2 h post-induction with 25 μM cumate. Values are given as mean ±SD of three independent experiments.

The PhyT- and SdrG-dependent PhyR phosphorylation shown above implies that the Paks' main function *in vivo* is to phosphorylate SdrG rather than PhyR, although Paks phosphorylate PhyR *in vitro* in the presence of NepR [19]. To further investigate PhyT- and SdrG-dependent PhyR phosphorylation in stress-induced GSR activation *in vivo*, we determined EcfG activity using β-galactosidase reporter assays. We analyzed a *pakB-G* deletion mutant (thus leaving *pakA* intact), with and without an additional *sdrG* knockout pre- and post-induction with a chemical stress mixture. Although we observed residual GSR in the absence of SdrG, no increased GSR activation could be observed upon addition of the chemical stress mixture (Fig 3). Next, we tested GSR induction upon overexpression of *pakA* in an *sdrG* knockout background and a *pakA*-*G* deletion mutant with and without an additional *sdrG* knockout. We observed that overexpression of *pakA* bypasses the *sdrG* knockout (Fig 3), which is congruent with previous work [19]. This implies that PhyT- and SdrG-independent PhyR phosphorylation by the Paks is possible *in vivo*, but plays only a minor role under physiological expression levels of the *paks*. Additionally, considering that an *sdrG* knockout may lead to an artificial increase of direct PhyR phosphorylation by Paks, the observed residual GSR activation is likely to be even lower in wild-type cells.

We also tested stress-dependent GSR induction in a *pakA-G* deletion mutant. In this strain, the GSR was still inducible upon stress application (Fig 3), which points to an additional kinase present in *S*. *melonis* Fr1 leading to PhyR phosphorylation. In the light of this finding, we performed EcfG-dependent β-galactosidase assays emphasizing the dependency of GSR induction on both SdrG and PhyT. In a *pakA-G* deletion mutant, an additional *phyT* knockout abolished GSR induction under stress conditions, indicating that the so far unidentified kinase strictly depends on the phosphorelay involving PhyT and SdrG (S3A Fig). GSR inducibility was rescued upon overexpression of *phyT* in this background. However, overexpression of *phyT* did not enable GSR induction under stress conditions in a *pakA-G* deletion mutant when an additional *sdrG* knockout was introduced (S3A Fig). Importantly, overexpression of *sdrG* in a *pakA-G* deletion mutant with an additional *phyT* knockout did not increase GSR (S3B Fig), which is in-line with the dependency of SdrG and PhyT on each other and their positive regulatory role in GSR activation.

### The phosphotransferase PhyT forms a complex with PhyR at the onset of GSR activation

**Fig 4:**
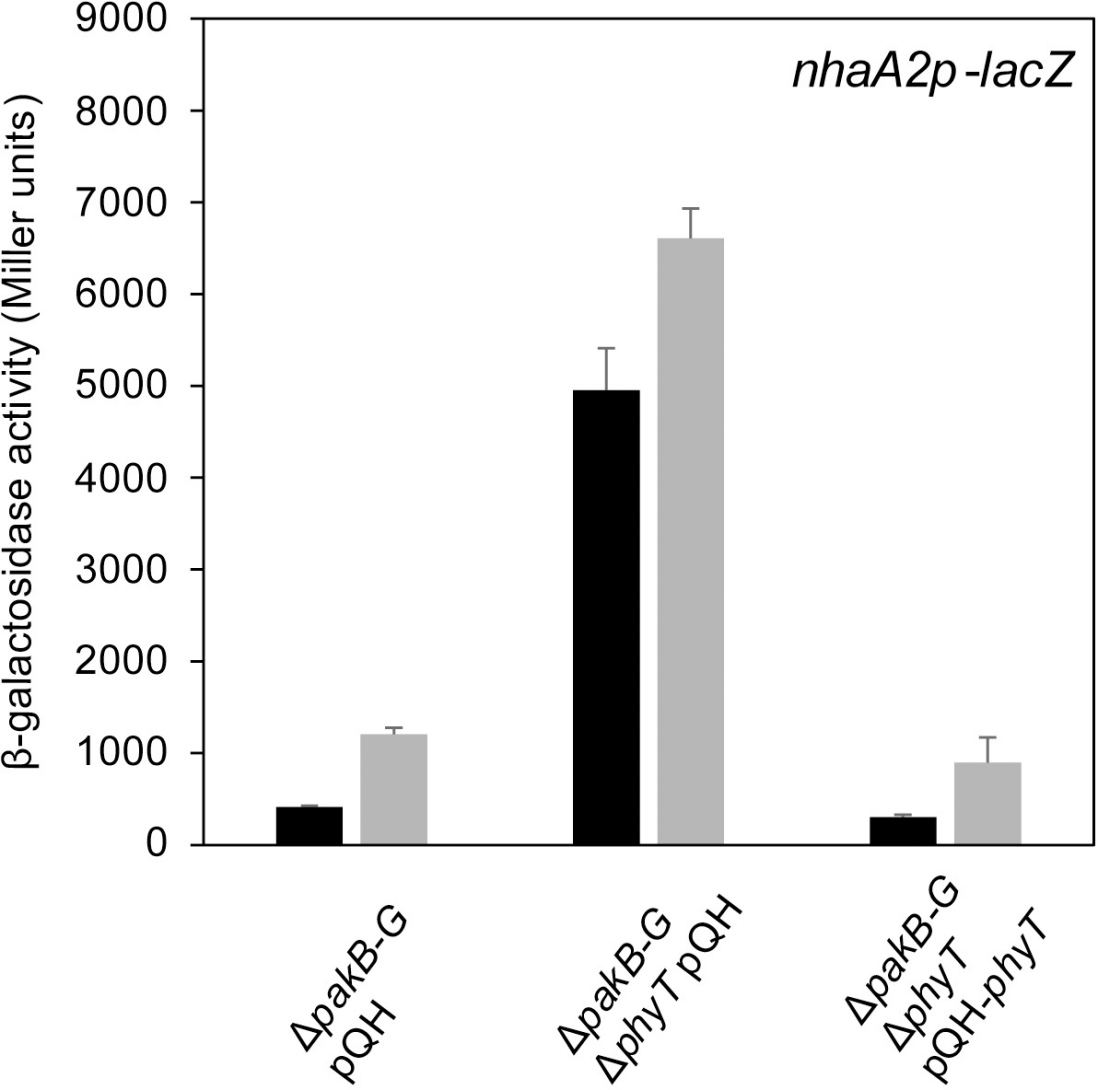
PhyT is a negative regulator of GSR *in vivo*. β-galactosidase activity of the EcfG-dependent *nhaA2p-lacZ* fusion in indicated *S*. *melonis* Fr1 mutant backgrounds upon overnight overexpression of *phyT* from the cumate-inducible pQH vector with 25 μM cumate. Empty pQH vector was used as a negative control. Black bars and gray bars represent β-galactosidase activity pre- and 1 h post-induction with the chemical stress mixture (1% ethanol, 80 mM NaCl and 50 μM TBHP). Values are given as mean ±SD of three independent experiments.

Because PhyT functions as a phosphotransferase (Fig 1), one would predict that it plays a role as a positive regulator of the GSR, which is puzzling in light of its described function as a negative regulator [14]. The latter function was deduced from the ability of PhyT to disrupt the NepR/PhyR complex *in vitro*—interpreted as phosphatase activity—and on the finding that its knockout is only possible in a GSR-impaired background, implying a lethal over activation of GSR in the absence of PhyT [14]. Based on these findings, we aimed to further characterize the negative regulatory role of PhyT. First, we performed EcfG-dependent β-galactosidase assays using a *pakB-G* deletion mutant with only *pakA* present. The knockout of *phyT* in this background resulted in increased GSR pre- and post-induction with a chemical stress mixture (Fig 4), which was caused by direct PakA-dependent PhyR phosphorylation. Overexpression of *phyT* complemented the observed phenotype (Fig 4). This result confirms that PhyT performs an additional negative GSR regulatory function *in vivo*.

**Fig 5:**
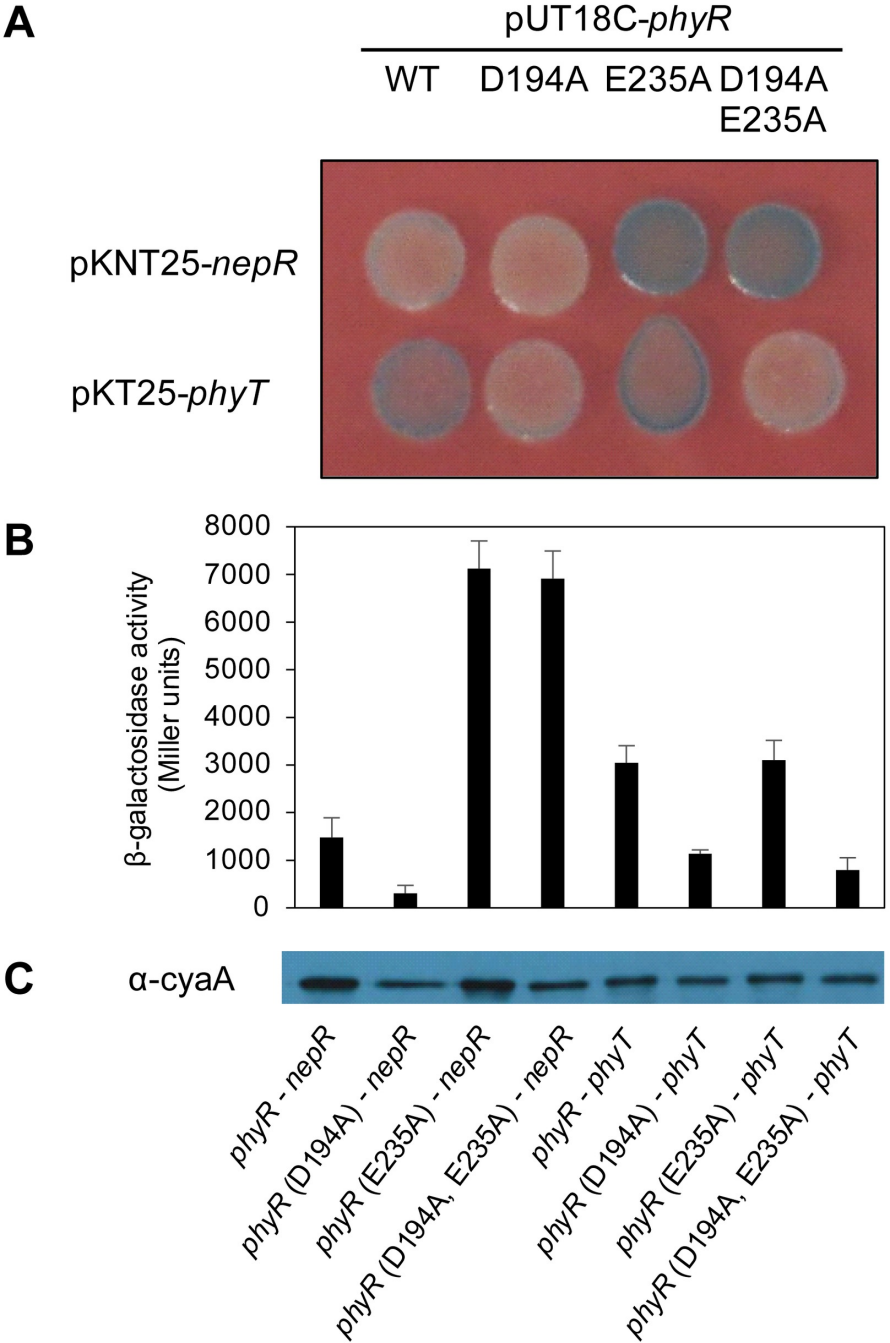
PhyT forms a complex with PhyR. (A) BACTH assay with bacteria spotted onto LB plates containing X-Gal (40 μg/mL), IPTG (0.5 mM) and antibiotics for selection. Wild-type PhyR and PhyR derivatives carrying either a D194A, a E235A or a combination of both mutations, were analyzed as C-terminal T18 fusions, while wild-type NepR was fused N-terminally and PhyT C-terminally to the T25 fragment of the *B*. *pertussis* CyaA protein to investigate interaction. Pictures were taken after 24 h of incubation at 30°C. Blue colonies indicate protein interaction. This image is a representative of three independent experiments. (B) β-galactosidase assays were performed for quantification in three biological replicates. Overnight cultures containing 0.5 mM IPTG and antibiotics for selection, were inoculated from single colonies of the co-transformed bacteria and incubated at 30°C. (C) Adenylate cyclase T18 fusions to PhyR proteins were detected in the samples used for quantitative analysis with Western blot analysis with CyaA monoclonal antibody (3D1) (1:2.000) (Santa Cruz Biotechnology) and a goat α-mouse antibody (1:3.000) with an exposure time of 2 min.

We speculated that PhyT controls the amount of phosphorylated PhyR by binding the response regulator and thereby preventing its direct phosphorylation by Paks. To test this working model, we first performed a bacterial two-hybrid (BACTH) assay. We used wild-type PhyR and a derivative in which the phosphorylatable Asp-194 residue of the receiver domain was mutated. In addition, we also examined a PhyR Glu-235 mutant, which contains a mutation located at the interface of the sigma factor-like and the receiver domain and results in a constitutively active PhyR [20]. Binding of the response regulator derivatives to the anti-sigma factor NepR was validated as controls. We showed that, indeed, PhyR and PhyT interact in the BACTH assay and that PhyT forms a dimer using plate assays (Fig 5A and S4A Fig) as well as β-galactosidase assays (Fig 5B and S4B Fig). Interestingly, the D194A mutation in PhyR seems to weaken its interaction with PhyT, while no effect on PhyT interaction could be observed for the E235A derivative (Fig 5). However, we did not observe interactions between either PhyR and any of the Paks or between SdrG and PhyT (S4 Fig).

### PhyR sequestration to the membrane by PhyT and its release upon stress induction

**Fig 6:**
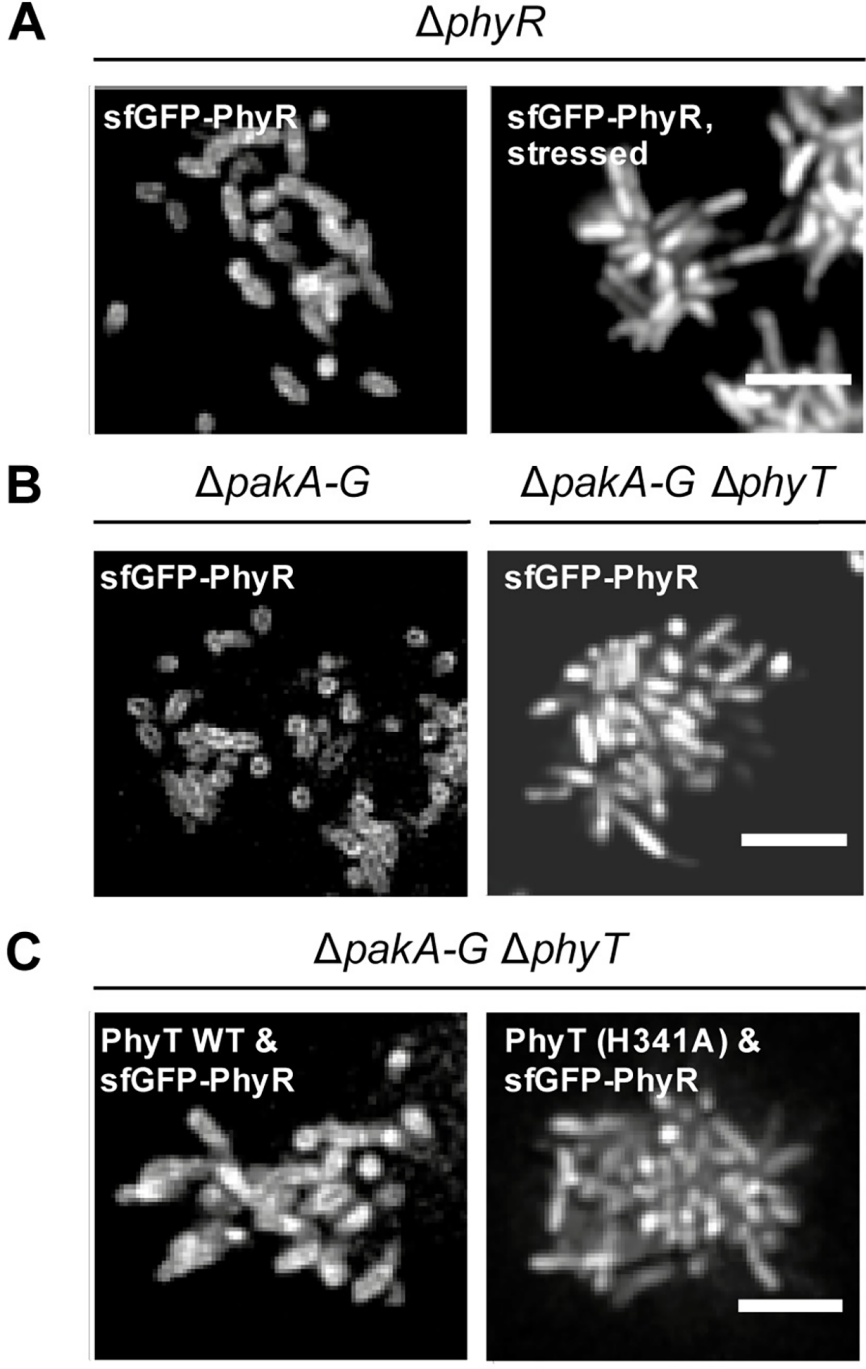
Membrane localization of PhyR depends on stress level in a H341-PhyT dependent fashion. Spinning-disc confocal images of different *S*. *melonis* Fr1 knockout mutants (A) upon production of sfGFP-PhyR, which was induced by addition of 25 μM cumate for 12 min. The chemical stress mixture (1% ethanol, 80 mM NaCl and 50 μM TBHP) was applied for 60 min. (B) Bacteria were imaged under unstressed conditions upon production of sfGFP-PhyR, which was induced by addition of 25 μM cumate for 12 min. (C) Bacteria were imaged under unstressed conditions upon overnight production of PhyT or the PhyT (H341A) derivative, which was induced by addition of 25 μM cumate and production of sfGFP-PhyR, induced by addition of 250 μM vanillate for 12 min. Scale bar, 5 μm. Comparable production of PhyT and the PhyT (H341A) derivative was confirmed using Western blot analysis (S2C Fig).

The protein-protein interaction of PhyR and PhyT predicts that PhyR is bound to the membrane under unstressed conditions. To test this, we studied the interaction between PhyR and PhyT by observing the association of sfGFP-tagged PhyR to the cell membrane depending on the stress condition and the presence/absence of PhyT using fluorescence microscopy. In a first set of experiments, we found that PhyR localized to the membrane under unstressed conditions, which supports binding of PhyR to the membrane-anchored PhyT (Fig 6A), while a homogeneous distribution was observed for sfGFP alone (S5 Fig). PhyR dissociated from the membrane under stress conditions, presumably due to PhyR phosphorylation and subsequent binding to NepR (Fig 6A). To confirm that PhyT is required for PhyR membrane localization, the cellular distribution of sfGFP-tagged PhyR was examined in a *pakA-G* deletion mutant with and without an additional *phyT* knockout. Supporting our previous results, membrane association of PhyR was abolished in the absence of PhyT (Fig 6B). Our data imply that PhyT binds to unphosphorylated PhyR, which is in agreement with the BACTH assay (Fig 5).

In addition, we tested the relevance of the phosphorylatable His-341 of PhyT for the interaction with PhyR (Fig 6C). We observed membrane localization of sfGFP-PhyR in a *pakA-G* deletion mutant with an additional *phyT* knockout mutant overproducing wild-type PhyT, while overproduction of the PhyT (H341A) derivative in the same strain background led to a homogeneous distribution of sfGFP-PhyR in the cells (Fig 6C).

Taken together, our results support complex formation between PhyT and PhyR. Furthermore, we showed that PhyR is released upon stress induction, indicating that only unphosphorylated PhyR is bound to PhyT, which provides a means by which PhyR is sequestered from direct phosphorylation by the Paks.

## Discussion

**Fig 7:**
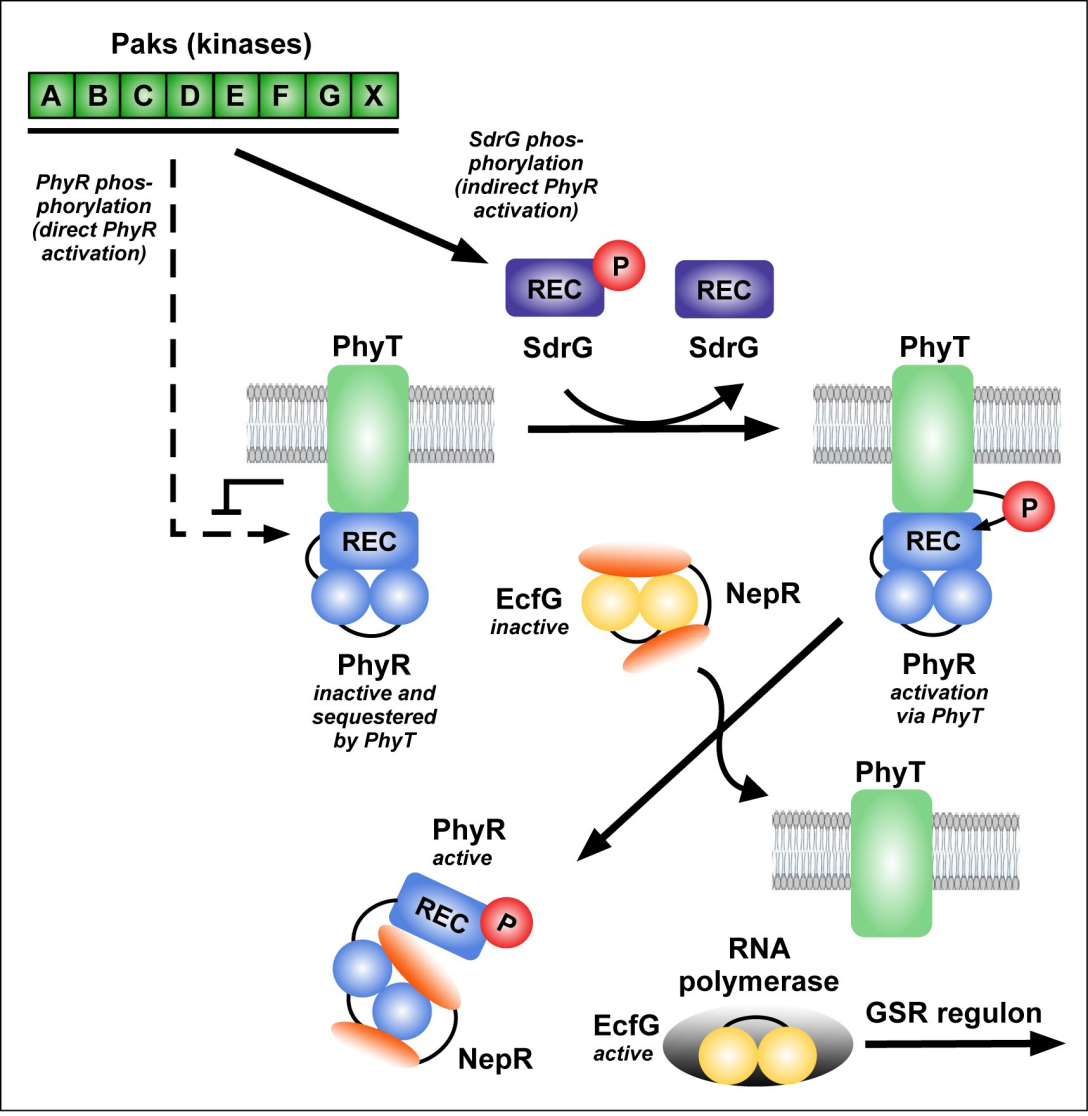
Model for GSR regulation in *S*. *melonis* Fr1, which involves SdrG and PhyT. Upon stress induction, the Paks autophosphorylate and transfer the phosphoryl group to the SDRR SdrG. Direct phosphorylation of PhyR by the Paks is inhibited due to PhyT-PhyR complex formation. PhyT transfers the phosphoryl group from SdrG~P to PhyR. PhyR~P dissociates from PhyT to bind the anti-sigma factor NepR and thereby releases the alternative sigma-factor EcfG, which initiates transcription of the GSR regulon by binding to RNA polymerase. For further details on the PhyR-NepR-EcfG cascade and Paks see [19, 21].

In this study, we uncovered essential functions in the regulation of the GSR in the alphaproteobacterium *S*. *melonis* Fr1, which resulted in an extended model of the core pathway involved in the activation of the crucial regulon (Fig 7). Based on our data, we defined the function of membrane-anchored PhyT, formerly described as the PhyR phosphatase PhyP, [14] and placed the SDRR SdrG in the core mechanism of GSR. We present *in vivo* and *in vitro* evidence that the negative regulatory function of PhyT, which prevents the lethal over activation of the GSR [14], relies on complex formation with unphosphorylated PhyR (Figs 5 and 6). Our results also identify PhyT as a phosphotransferase participating in a GSR-activating phosphorelay (Fig 1). The importance of the Pak-SdrG-PhyT-PhyR phosphorelay became evident when we discovered that both SdrG and PhyT are needed for appropriate GSR activation in stressful conditions *in vivo* (Figs 2 and 3, S3 Fig).

Altogether, we suggest a model for GSR activation according to which Paks phosphorylate SdrG upon stress induction. When the concentration of SdrG~P reaches a threshold level, it induces the phosphotransferase activity of PhyT in a competitive way, thereby temporarily replacing PhyR to transfer its phosphoryl group to PhyT. EcfG-bound NepR could then eventually prime PhyR [35] via direct interaction for facilitated phosphorylation by PhyT. Overall, our data suggest a mechanism by which a precise interplay between the bifunctional regulator PhyT, SdrG, PhyR and the seven stress-sensing Paks is essential to ensure appropriate GSR induction via "sigma factor mimicry" and thus via NepR and the general stress sigma factor EcfG.

Our *in vitro* data indicate that the phosphotransferase PhyT irreversibly transfers the phosphoryl group from SdrG~P to PhyR (Fig 1 and S1 Fig). This is in contrast to other described bacterial phosphotransferases such as Spo0B of *B*. *subtilits* that catalyze reversible phosphotransfer reactions [36]; however, note that unidirectional phosphotransfer has been observed for the histidine phosphotransferase YPD-1 in *Saccharomyces cerevisae* to the response regulator SSK1-R2 [37]. Currently, we cannot rule out that PhyT switches its activity to PhyR dephosphorylation *in vivo* either by turning into a phosphatase or by catalyzing the reverse phosphotransfer reaction in response to external signals e.g. via its so far uncharacterized periplasmic domain or via protein-protein interaction with so far unknown interaction partners. It thus remains to be investigated how the GSR is shut down and whether an additional phosphatase exists. Additionally, it is unknown whether the system relies on the instability of the phosphoryl-Asp of PhyR, basal PhyR protein turnover or if stress-dependent PhyR degradation plays a role, as it has been observed in *B*. *abortus* [38].

*S*. *melonis* Fr1 inhabits the phyllosphere, a stressful environment characterized by constantly changing conditions [13]. Therefore, it is likely that a strict regulatory system such as the GSR is under constant pressure to evolve in terms of sensitivity and specificity. PhyT and SdrG might have co-evolved in *S*. *melonis* Fr1 in order to ensure appropriate GSR induction while simultaneously counteracting the danger of lethal over activation.

We suspect that PhyT evolved from a histidine kinase, e.g. via degeneration of its catalytic domain [9, 14]. Precedence exists in other two-component systems that involve degenerated histidine kinases. These include the dimeric histidine phosphotransferases Spo0B from *B*. *subtilis* involved in the initiation of sporulation [5] and ChpT from *C*. *crescentus*, which is involved in cell cycle progression [6, 39, 40]. Notably, the evolution of PhyT-type regulators is not limited to the model strain *S*. *melonis* Fr1, but rather conserved among alphaproteobacteria [9]. Various studies have been conducted on PhyT-type negative GSR regulators [11, 17, 18, 31], albeit the mechanism by which they act at the molecular level remains to be elucidated. It is conceivable that this protein functions as a phosphotransferase in other alphaproteobacteria as well. Nonetheless, features and regulatory strategies of the regulators mediating the GSR seem to have diversified to different extents in the phylogenetic class, likely as a result of gene duplication events and divergent evolutionary pressures in the various linages of alphaproteobacteria. Regardless, GSR regulation must be precisely coordinated with the requirements of the corresponding bacterium, i.e. depending on the properties of the present stress-sensing kinases in terms of specificity and efficiency regarding PhyR phosphorylation. Thereby, an increasing number of partially redundant kinases as in *S*. *melonis* Fr1 [19] and *M*. *extorquens* [27] might bring the need to build in tunable negative regulators and a phosphorelay. Altogether, the characterization of PhyT together with SdrG as essential players in GSR regulation in *S*. *melonis* Fr1 shades light on an evolutionary pattern of histidine kinase derived regulators, which is conserved within and beyond the boundaries of the GSR.

## Methods

### Mutant strain construction

*S*. *melonis* Fr1 in-frame deletion mutants were constructed with the plasmid pAK405 via double homologous recombination [41].

### Plasmid construction

All plasmids constructed during this study are listed in S1 Table. The primers used for plasmid construction are listed in S2 Table.

#### Construction of knockout plasmids

Up- and downstream regions (~750 bp) of the respective genes were amplified with PhyR HR1 fwd (KpnI)/ PhyR HR1 rev and PhyR HR2 fwd/PhyR HR2 rev (HindIII) for *phyR* and with HR1 PhyT fwd (BamHI)/HR1 PhyT rev and HR2 PhyT fwd/HR2 PhyT rev (HindIII) for *phyT* from genomic DNA of *S*. *melonis* Fr1. PCR products were joined with an overlap PCR and cloned into pAK405 [41] with KpnI/HindIII for *phyR* and BamHI/HindIII for *phyT*.

#### Construction of plasmids for bacterial two-hybrid assays

*NepR*, *phyT*, *sdrG* or *ecfG* were amplified with the primers NepR fwd/NepR rev, PhyT fwd/PhyT rev, SdrG fwd/SdrG rev or EcfG fwd/EcfG rev from genomic DNA of *S*. *melonis* Fr1. After digestion with XbaI/KpnI, the fragments were inserted into pUT18, pUT18C or pKT25 [42]. Site-directed mutagenesis of Asp-194 or Glu-235 of PhyR encoded on pUT18C-*phyR* was carried out with the primers PhyR D194A_fwd/PhyR D194A_rev and PhyR E235A_fwd/PhyR E235A_rev.

#### Construction of expression plasmids for fluorescence microscopy

*sfGFP* was amplified from pTE100-*sfGFP* with the primers sfGFP fwd (HindIII)/sfGFP rev (AseI), and after digestion with HindIII and AseI, was ligated into pQY [43] or pQYD-*phyR*, thereby replacing s*YFP2*. Cloning resulted in the cumate-inducible expression plasmids pQY-*sfGFP* and pQYD-*sfGFP*-*phyR*. *sfGFP*-*phyR* was amplified from pQYD-*sfGFP*-*phyR* with the primers *sfGFP*-*phyR* fwd (HindIII)/*sfGFP*-*phyR* rev (XhoI). After digestion with HindIII and XhoI, the fragment was ligated into the vanillate-inducible expression plasmid pVH, thereby replacing the N-terminal HA-tag encoded on the plasmid. Cloning resulted in the vanillate-inducible expression plasmid pVH-*sfGFP*-*phyR*. For site-directed mutagenesis of His-341 into Ala in *phyT* encoded on the cumate-inducible pAK200-*phy*T plasmid, the primers PhyT H341A fwd/PhyT H341A rev were used, resulting in pAK200-*phyT* (H341A).

#### Construction of SdrG expression plasmid for *in vitro* phosphotransfer assays

*SdrG* was amplified from pET26bII-*sdrG-His*_*6*_ [19] with the primers SdrG fwd in pASK-IBA3/SdrG rev in pASK-IBA3. The PCR product was digested with BamHI/NcoI and ligated into pASK-IBA3 to obtain pASK-IBA3-*sdrG*-Strep. In this construct, the Strep-tag and *sdrG* are connected with a thrombin cleavage site. From this plasmid, *sdrG*-Strep was amplified with the primers SdrG_Strep (HindIII) rev/SdrG_Strep (PciI) fwd. The PCR product was digested with HindIII/PciI and cloned into pET28b to obtain pET28b-*sdrG*-Strep for IPTG-inducible heterologous expression in *E*. *coli*.

#### Site-directed mutagenesis for SdrG variants

For site-directed mutagenesis of Asp-56 in *sdrG* encoded on the vanillate-inducible pVH backbone [44], the primers SdrG D56E fwd/SdrG D56E rev were used for mutation to Glu and SdrG D56A fwd/SdrG D56A rev were used for mutation to Ala.

### Protein expression and purification

For heterologous expression of NepR, PhyR, and SdrG in *E*. *coli*, overnight cultures of BL21(DE3)Gold pET26bII-*nepR-His*_*6*_, pET26bII-*phyR-His*_*6*_ and pET28b-*sdrG-Strep* (S1 Table) were grown in 5 mL LB-Lennox medium supplemented with kanamycin (50 μg/mL). Stationary cultures were diluted and incubated further at 37°C to an OD600 of 0.8. After induction with 1 mM IPTG, cells were grown for 3.5 h at 37°C prior to harvest. The pellets were washed once with 1x cold PBS before being stored at -80°C. For expression of PakF, a BL21-Gold (DE3) pDEST-*His*_*6*_*-MBP*-*pakF* pre-culture was inoculated in the morning in LB-Lennox medium supplemented with carbenicillin (50 μg/mL) and grown until turbidity could be observed. The main culture was subsequently inoculated and incubated at 37°C. As soon as an OD600 of 0.8 was reached, the flask was placed on ice for 30 min. Afterwards, expression was induced with 50 μM IPTG and further incubation overnight was conducted at 16°C. Cells were washed once with 1x PBS prior to harvest and stored at -80°C. NepR-, PhyR-, and PakF-containing cell pellets were thawed and resuspended in lysis buffer (20 mM HEPES, 0.5 M NaCl, 10% glycerol, 1 tablet protease inhibitor (EDTA-free, Roche), 2 mM beta-mercaptoethanol, 0.1 mg/mL DNase and 20 mM imidazole; pH 8.0). Cells were passed 3x through the French press for cell lysis, followed by centrifugation (12.000g, 30 min, 4°C) to remove cell debris. The supernatant was incubated for 1 h at 4°C with 500 μL Ni-NTA beads (Macherey-Nagel) (1 mL slurry, washed 2x with wash buffer (20 mM HEPES, 0.5 M NaCl, 10% glycerol and 20 mM imidazole; pH 8.0)). Afterwards, the beads were loaded on a polypropylene column. Proteins were eluted from the Ni-NTA resin with 2.6 mL elution buffer (wash buffer with 200 mM imidazole) after washing with 40 mL wash buffer by gravity flow. The SdrG-containing cell pellet was thawed and also resuspended in imidazole-free lysis buffer and passed 3x through the French press. After centrifugation, SdrG-containing supernatant was added to a polypropylene column loaded with 1 mL Strep-tactin beads (IBA Lifesciences) (2 mL slurry), which were equilibrated with 2 mL imidazole-free wash buffer prior to use. The beads were washed with 10 mL imidazole-free wash buffer by gravity flow, before SdrG-Strep was eluted with 3 mL wash buffer supplemented with 2.5 mM desthiobiotin. All cleaned up proteins were subjected to PD10 desalting columns for exchange to kinase buffer (10 mM HEPES, 50 mM KCl, 10% glycerol; pH 8.0). The purified proteins were concentrated with 3 kDa cutoff amicon tubes (Millipore). Protein concentrations were determined with a BCA protein assay (Life Technologies Europe B.V.). 50 μL aliquots were stored at -20°C.

### Preparation of *E*. *coli* membrane particles

Heterologous overexpression of PhyT and the PhyT (H341A) derivative was carried out as described for NepR and PhyR using IPTG-inducible pET26bII expression plasmids (S1 Table). Cell pellets were thawed and resuspended in imidazole-free lysis buffer. Cells were passed 3x through the French press. The supernatant of the first centrifugation step (12.000g, 30 min, 4°C) was subjected to ultracentrifugation (180.000g, 1 h, 4°C). The membrane pellets were resuspended in kinase buffer to a concentration of 100 mg membrane fraction/mL and 100 μL aliquots were stored at -80°C. Comparable amounts of PhyT and the PhyT (H341A) derivative in the prepared *E*. *coli* membrane particles were confirmed with standard SDS-PAGE followed by Western blot analysis using a mouse Tetra·His antibody (1:2.000) (34570 Qiagen AG) and a goat α-mouse HPR-coupled antibody (1:3.000) (BioRad) (S2A and S2B Fig).

### *In vitro* phosphotransfer reactions

Protein expression and purification as well as preparation of *E*. *coli* membrane particles are described above. Purified proteins were thawed on ice. First, PakF was diluted to a concentration of 10 μM in kinase buffer supplemented with 10 mM MgCl_2_ and 1 mM DTT. 200 μL Ni-NTA beads (400 μL slurry) were washed 2x with kinase buffer supplemented with 10 mM MgCl_2_ and 1 mM DTT before 320 μL PakF (10 μM) were added and incubated at room temperature (RT) for 30 min. Afterwards, autophosphorylation of PakF was initiated with 3.2 μL [γ-^32^P]ATP (5.000 Ci/mmol; Hartmann Analytic GmbH). After 10 min of autophosphorylation, PakF bound to Ni-NTA resin was loaded on polypropylene columns and washed with kinase buffer until radioactivity of the wash fraction decreased significantly.

#### Phosphotransfer reactions

SdrG was diluted to 10 μM, PhyR to 50 μM, and NepR to 75 μM in kinase buffer supplemented with 10 mM MgCl_2_ and 1 mM DTT. The reaction mixtures were prepared in a total volume of 50 μL with PhyR (5 μM), NepR (7.5 μM), and PhyT or PhyT (H341A) (5 mg membrane fraction/mL) and kept on ice; however, they were allowed to adjust to RT 10 min prior to the phosphotransfer reaction. Next, the columns loaded with autophosphorylated PakF bound to Ni-NTA resin were closed with a screw cap, before 350 μL SdrG (10 μM) equilibrated to RT were added. After 60 seconds, SdrG~P was allowed to elute from the columns. To start the phosphotransfer reaction, 50 μL of SdrG~P were added to 50 μL of the reaction mixtures. After 0.5 min, 1 min, 3 min, 5 min and 10 min, 10 μL of the reaction were withdrawn and quenched by mixing with 3x Lämmli buffer.

#### Dephosphorylation reactions

SdrG was diluted to a concentration of 50 μM, PhyR to 10 μM, and NepR to 15 μM in kinase buffer supplemented with 10 mM MgCl_2_ and 1 mM DTT. Reaction mixtures were prepared in a total volume of 10 μL with SdrG (5 μM) and PhyT or PhyT (H341A) (5 mg membrane fraction/mL) and kept on ice. The mixtures were allowed to adjust to RT 10 min prior to the phosphotransfer reaction. 100 μL PhyR and 100 μL NepR solutions were mixed and incubated for at least 10 min at RT. Next, 100 μL of the mixture were added to the column loaded with autophosphorylated PakF bound to Ni-NTA resin. On-column phosphorylation of PhyR in the presence of NepR was allowed to proceed for 2 min. After elution, the procedure was repeated. The aliquot with the highest detectable radioactivity was used for the dephosphorylation reaction. To start the reaction, 10 μL of phosphorylated PhyR/NepR mix were added to the reaction mixtures. After 60 sec, 10 μL of the reaction were withdrawn and quenched by mixing with 3x Lämmli buffer.

Subsequent to phosphotransfer and dephosphorylation reactions, the samples were subjected to 15% SDS-PAGE. The gel front, which might contain residual [γ-^32^P] ATP was removed before the gel was dried. For signal detection, a phosphoimager screen was exposed to the dried gel for 24–72 h, depending on the signal strength. Scanning of the screens was done with the FX Imager Pro Plus and the QuantityOne software (BioRad).

### β-galactosidase assays

*S*. *melonis* Fr1 strains carrying the reporter plasmid pAK501-*nhaA2p-lacZ* and an inducible vanillate or cumate expression plasmid (S1 Table) were streaked out from a cryo-stock on TYE agar plates (1% tryptone, 0.5% yeast extract, 2.4 mM Na_2_HPO_4_, 37.6 mM KH_2_PO_4_) containing tetracycline (10 μg/mL) and chloramphenicol (34 μg/mL) and incubated overnight at 28°C. The plates were sealed with parafilm and protected from light, to reduce stress exposure. Pre-cultures were inoculated from a small loop in 20 mL TYE medium supplemented with tetracycline (10 μg/mL) and chloramphenicol (34 μg/mL) in 100 mL baffled flasks and incubated at 28°C, 160 rpm during the day.

#### Overexpression of *sdrG* and *phyT*

50 mL overnight cultures were inoculated in TYE medium supplemented with tetracycline (10 μg/mL) and chloramphenicol (34 μg/mL) in 250 mL baffled flasks with either 25 μM cumate (100 mM stock in 100% ethanol) for expression of *phyT* or 250 μM vanillate (250 mM stock in 100% ethanol) for expression of *sdrG* and inoculated so as to obtain exponentially growing cells at the time point of the assay. When the cultures reached an OD600 of about 0.3–0.4, the 50 mL were divided in 2x 20 mL cultures and transferred into 100 mL baffled flasks. One of the two cultures was induced with the chemical stress mixture (1% ethanol, 80 mM NaCl, 50 μM *tert*-Butyl hydroperoxide (TBHP); Sigma-Aldrich). Measurement of the β-galactosidase activity (Miller 1972) was performed 1 h after stress induction.

#### Overexpression of *pakA*

20 mL overnight cultures were inoculated in TYE medium supplemented with tetracycline (10 μg/mL) and chloramphenicol (34 μg/mL) in 100 mL baffled flasks so that exponential growth was reached at the time point of the assay the next day. The first measurement was performed before addition of the chemical stress mixture, which was immediately added after samples were taken from the cultures for the first measurement. The second measurement was performed 1 h after stress induction. 25 μM cumate were added immediately after samples were taken to induce expression of *pakA*. The third measurement was performed 3 h post-induction with the chemical stress mixtures stress and 2 h post-induction with cumate.

### Bacterial two-hybrid assays

To test protein-protein interactions, C- and N-terminal translational fusions of the T18 and T25 domains of the adenylate cyclase CyaA of *Bordetella pertussis* [42] were used for PhyT, NepR, PhyR wild-type, PhyR derivatives and PakA-G (S1 Table). Fusions were tested in pairwise combinations in *E*. *coli* BTH101 *cya*^*-*^ (Euromedex). For the interaction analysis the optimal pair for each protein combination is shown. For each transformation mixture, 5 μl were spotted onto LB agar plates containing 0.5 mM isopropyl 1-thio-β-D-galactopyranoside and 40 μg/ml 5-bromo-4- chloro-3-indolyl β-D-galactopyranoside (X-Gal) with selection for carbenicillin (50 μg/mL) and kanamycin resistance (50 μg/mL). Plates were incubated at 30°C for 24 h. Formation of blue colonies was scored as a positive interaction. In addition, a volume of 20 μl of each transformation mixture was plated on LB agar plates containing carbenicillin (50 μg/mL) and kanamycin (50 μg/mL) for selection. Plates were incubated at 28°C overnight. For quantitative analysis, 5 mL cultures of LB supplemented with carbenicillin (50 μg/mL) and kanamycin (50 μg/mL) for selection and 0.5 mM IPTG were inoculated from single colonies. Overnight cultures were grown at 30°C and used to measure β-galactosidase activity (Miller 1972). 500 μl of bacterial culture were spun down and pellets were resuspended in 1x Lämmli buffer so that a final OD_600_ of 10 was reached. The samples were subjected to standard SDS-PAGE followed by Western blot analysis. For detection of T18 fusions the mouse α-CyaA (3D1) monoclonal antibody (Santa Cruz Biotechnology) (1:2.000) and a goat α-mouse HPR-coupled antibody (1:3.000) were used (BioRad).

### Fluorescence microscopy

*S*. *melonis* Fr1 strains carrying either pQY-*sfGFP* or pQYD-*sfGFP*-*phyR* (S1 Table) were streaked out from cryo-stock on TYE agar plates supplemented with tetracycline (10 μg/mL) and were incubated overnight at 28°C. The plates were sealed with parafilm and protected from light to avoid stress induction. Pre-cultures were inoculated in 20 mL TYE medium supplemented with tetracycline (10 μg/mL) in 100 mL baffled flasks and incubated during the day at 180 rpm and 28°C. Overnight cultures were inoculated at an OD600 appropriate to reach exponential growth phase at the time of the experiment. Incubation conditions were the same as for the pre-cultures. PhyR-sfGFP or sfGFP expression in the *S*. *melonis* Fr1 mutants was induced in mid-exponential phase via the addition of 25 μM cumate (100 mM stock in 100% ethanol) for 12 min. In order to analyze the importance of His-341 of PhyT for binding of PhyR and therefore membrane localization of sfGFP-PhyR, the appropriate strains carrying pVH-*sfGFP*-*phyR* and either pAK200-*phyT* or pAK200-*phyT* (H341A) (S1 Table) were streaked out from cryo-stock on TYE agar plates supplemented with tetracycline (10 μg/mL) and kanamycin (50 μg/mL). Following overnight incubation at 28°C, pre-cultures were inoculated in 20 mL TYE medium supplemented with tetracycline (10 μg/mL) and kanamycin (50 μg/mL) in 100 mL baffled flasks and cultivated as described above. In addition to both antibiotics, cumate (25 μM) was added to the 20 mL overnight cultures. Production of sfGFP-PhyR was induced on the following day with 250 μM vanillate (250 mM stock in 100% ethanol) for 12 min in mid-exponential phase. The bacteria were subsequently washed with Tris-buffered saline (50 mM Tris-HCl, 150 mM NaCl; pH 7.55) and mounted on a cover slip for imaging. The live-cell imaging was performed on an inverse spinning disc microscopy system (Visitron software, Yokogawa CSU-X1 spinning-disk confocal unit) equipped with a solid state Laser Unit (Toptica) at 488 nm excitation wavelength, a 100x Oil Plan-Neofluar Objective (Zeiss, NA: 1.3), and an Evolve 512 EMCCD camera (Photometrics). The images were deconvolved using Huygens Professional version 17.04 (Scientific Volume Imaging, The Netherlands, http://www.svi.nl/HuygensSoftware).

## Supporting information

S1 Fig No phosphatase activity of PhyT (formerly PhyP) on PhyR was observed *in vitro*. The histidine kinase PakF was allowed to autophosphorylate with [γ-32P] ATP following Ni-NTA binding. PhyR (5 μM) in the presence of NepR (7.5 μM) was phosphorylated by Ni-NTA-bound PakF. The dephosphorylation assay was started by adding the mixture of PhyR~P and NepR to the reaction mixtures containing combinations of *E*. *coli* membrane particles (5 mg membrane fraction/mL) harboring either wild-type PhyT or the PhyT (H341A) derivative and SdrG (5 μM). This image is a representative of two independent experiments. For confirmation of comparable amounts of PhyT and the PhyT (H341A) derivative, Western blot analysis was conducted (S2B Fig).
(TIF)

S2 Fig Western blots for protein expression control. (A) *E*. *coli* membrane particles were analyzed for the presence of PhyT wild-type or the PhyT (H341A) derivative produced from the IPTG-inducible expression plasmid pET26bII using a mouse Tetra·His antibody (1:2.000) and a goat α-mouse antibody (1:3.000) to ensure equal amounts of both proteins in the phosphotransfer reactions (Fig 1). (B) *E*. *coli* membrane particles were analyzed as in (A) for the presence of PhyT and the PhyT (H341A) derivative to ensure comparable amounts of both proteins in the dephosphorylation assay (S1 Fig). (C) Comparable production of PhyT wild-type and the PhyT (H341A) derivative from the cumate-inducible pAK200 expression plasmid used for the sfGFP-PhyR membrane localization study (Fig 6C) was tested using a mouse α-Flag antibody (1:2.000) and a goat α-mouse antibody (1:3.000). (D) & (E) Adenylate cyclase T18-fusion proteins were detected in the samples used for quantitative analysis of the BACTH assay (S4 Fig) with Western blot analysis using a mouse α-CyaA monoclonal antibody (3D1) (1:2.000) (Santa Cruz Biotechnology) and a goat α-mouse antibody (1:3.000). Exposure time were 30 sec for (A), 20 sec for (B), 2 min for (C), 4 min for (D) and 1 min for (E).
(TIF)

S3 Fig PhyT and SdrG are both important *in vivo* for GSR induction. β-galactosidase activity of the EcfG-dependent *nhaA2p-lacZ* fusion in indicated *S*. *melonis* Fr1 mutant backgrounds (A) upon overnight overexpression of *phyT* from the cumate-inducible pQH vector with 25 μM cumate. Empty pQH vector was used as a negative control. (B) Overnight overexpression of *sdrG* from vanillate-inducible pVH vector with 250 μM vanillate. pVH only was used as empty-vector control. Black bars and gray bars represent β-galactosidase activity pre- and 1 h post-induction with the stress mixture (1% ethanol, 80 mM NaCl and 50 μM TBHP). Values are given as mean ±SD of three independent experiments.
(TIF)

S4 Fig PhyR does not interact with the Paks in BACTH assays. (A) BACTH assay with bacteria spotted onto LB plates containing X-Gal (40 μg/mL), IPTG (0.5 mM), and antibiotics for selection. Interactions of the C-terminal T18-PhyR wild-type with N-terminal T25-Pak fusions were tested. Stable interaction between N-terminal T18-NepR fusion and C-terminal T25-EcfG fusion was confirmed as a control. PhyT dimerization was shown with C-terminal fusion proteins. SdrG-PhyT interaction was tested with C-terminal T18-SdrG and C-terminal T25-PhyT fusion proteins. Pictures were taken after 24 h of incubation at 30°C. Blue colonies indicate protein interaction. (B) β-galactosidase assays were performed for quantification in three biological replicates. Overnight cultures containing 0.5 mM IPTG and antibiotics for selection, were inoculated from single colonies of the co-transformed bacteria and incubated at 30°C.
(TIF)

S5 Fig sfGFP does not localize to the membrane in *S*. *melonis* Fr1. Spinning-disc confocal image of the *S*. *melonis* Fr1 Δ*phyR* knockout mutant upon production of sfGFP, which was induced by addition of 25 μM cumate for 12 min. Scale bar, 5 μm.
(TIF)

S1 Table Plasmids and strains. (DOCX)

S2 Table Primers for plasmid construction and site-directed mutagenesis. (DOCX)
