# Supplementary figures and images for: Phosphorelay through the bifunctional phosphotransferase PhyT controls the general stress response in an alphaproteobacterium
Source: PLoS Genet. 2018 Apr 13;14(4):e1007294. doi: 10.1371/journal.pgen.1007294 (PMC5898713; doi:10.1371/journal.pgen.1007294)

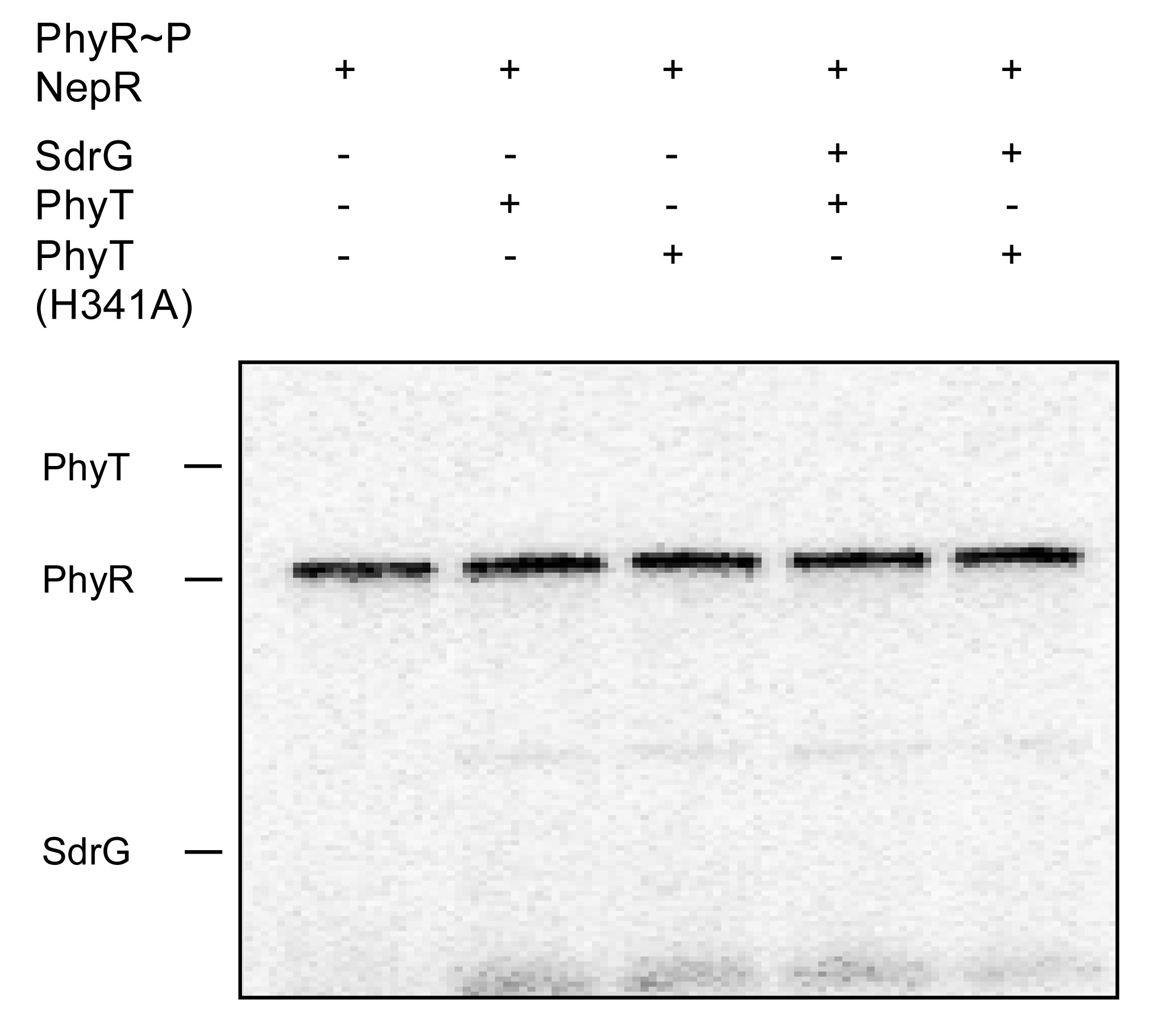

Supplement: S1 Fig — The histidine kinase PakF was allowed to autophosphorylate with [γ-32P] ATP following Ni-NTA binding. PhyR (5 μM) in the presence of NepR (7.5 μM) was phosphorylated by Ni-NTA-bound PakF. The dephosphorylation assay was started by adding the mixture of PhyR~P and NepR to the reaction mixtures containing combinations of E. coli membrane particles (5 mg membrane fraction/mL) harboring either wild-type PhyT or the PhyT (H341A) derivative and SdrG (5 μM). This image is a representative of two independent experiments. For confirmation of comparable amounts of PhyT and the PhyT (H341A) derivative, Western blot analysis was conducted (S2B Fig). (TIF) [file pgen.1007294.s001.tif]

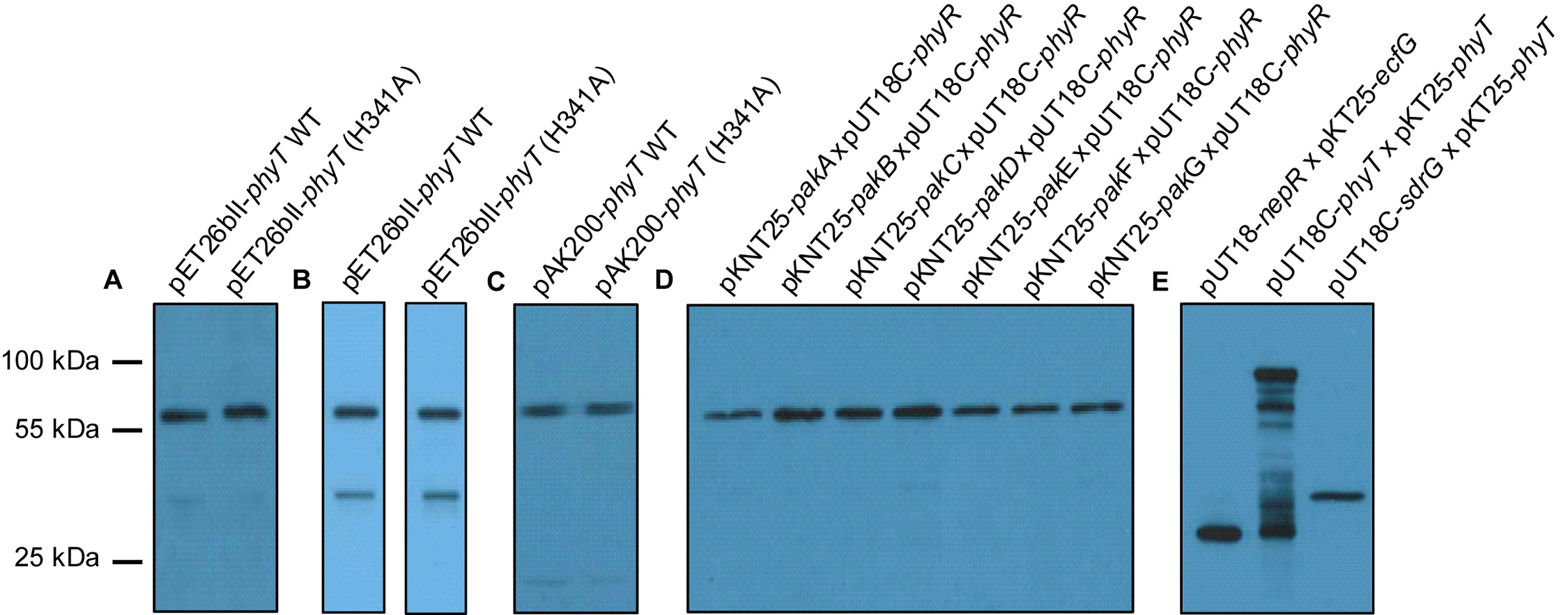

Supplement: S2 Fig — (A) E. coli membrane particles were analyzed for the presence of PhyT wild-type or the PhyT (H341A) derivative produced from the IPTG-inducible expression plasmid pET26bII using a mouse Tetra·His antibody (1:2.000) and a goat α-mouse antibody (1:3.000) to ensure equal amounts of both proteins in the phosphotransfer reactions (Fig 1). (B) E. coli membrane particles were analyzed as in (A) for the presence of PhyT and the PhyT (H341A) derivative to ensure comparable amounts of both proteins in the dephosphorylation assay (S1 Fig). (C) Comparable production of PhyT wild-type and the PhyT (H341A) derivative from the cumate-inducible pAK200 expression plasmid used for the sfGFP-PhyR membrane localization study (Fig 6C) was tested using a mouse α-Flag antibody (1:2.000) and a goat α-mouse antibody (1:3.000). (D) & (E) Adenylate cyclase T18-fusion proteins were detected in the samples used for quantitative analysis of the BACTH assay (S4 Fig) with Western blot analysis using a mouse α-CyaA monoclonal antibody (3D1) (1:2.000) (Santa Cruz Biotechnology) and a goat α-mouse antibody (1:3.000). Exposure time were 30 sec for (A), 20 sec for (B), 2 min for (C), 4 min for (D) and 1 min for (E). (TIF) [file pgen.1007294.s002.tif]

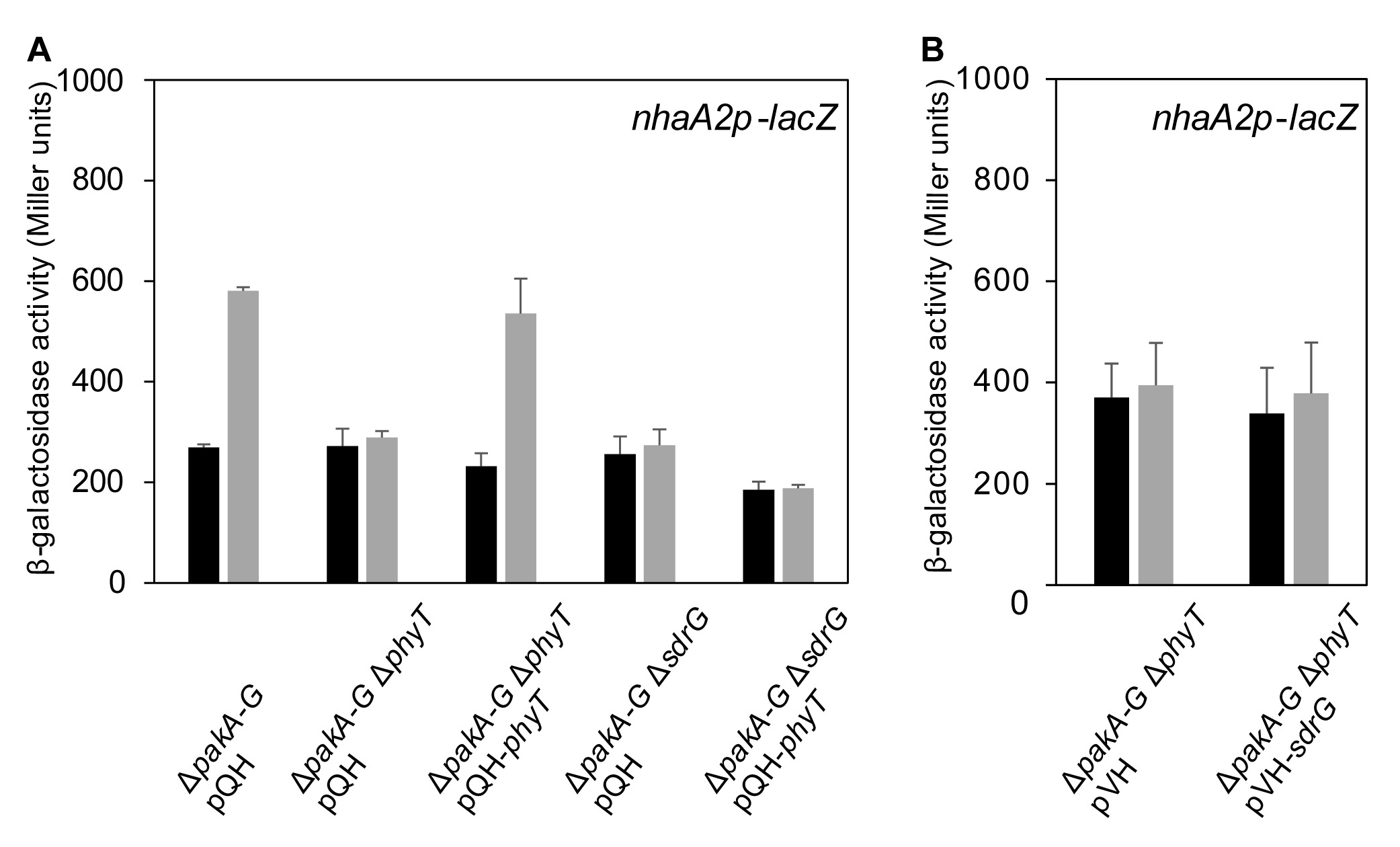

Supplement: S3 Fig — β-galactosidase activity of the EcfG-dependent nhaA2p-lacZ fusion in indicated S. melonis Fr1 mutant backgrounds (A) upon overnight overexpression of phyT from the cumate-inducible pQH vector with 25 μM cumate. Empty pQH vector was used as a negative control. (B) Overnight overexpression of sdrG from vanillate-inducible pVH vector with 250 μM vanillate. pVH only was used as empty-vector control. Black bars and gray bars represent β-galactosidase activity pre- and 1 h post-induction with the stress mixture (1% ethanol, 80 mM NaCl and 50 μM TBHP). Values are given as mean ±SD of three independent experiments. (TIF) [file pgen.1007294.s003.tif]

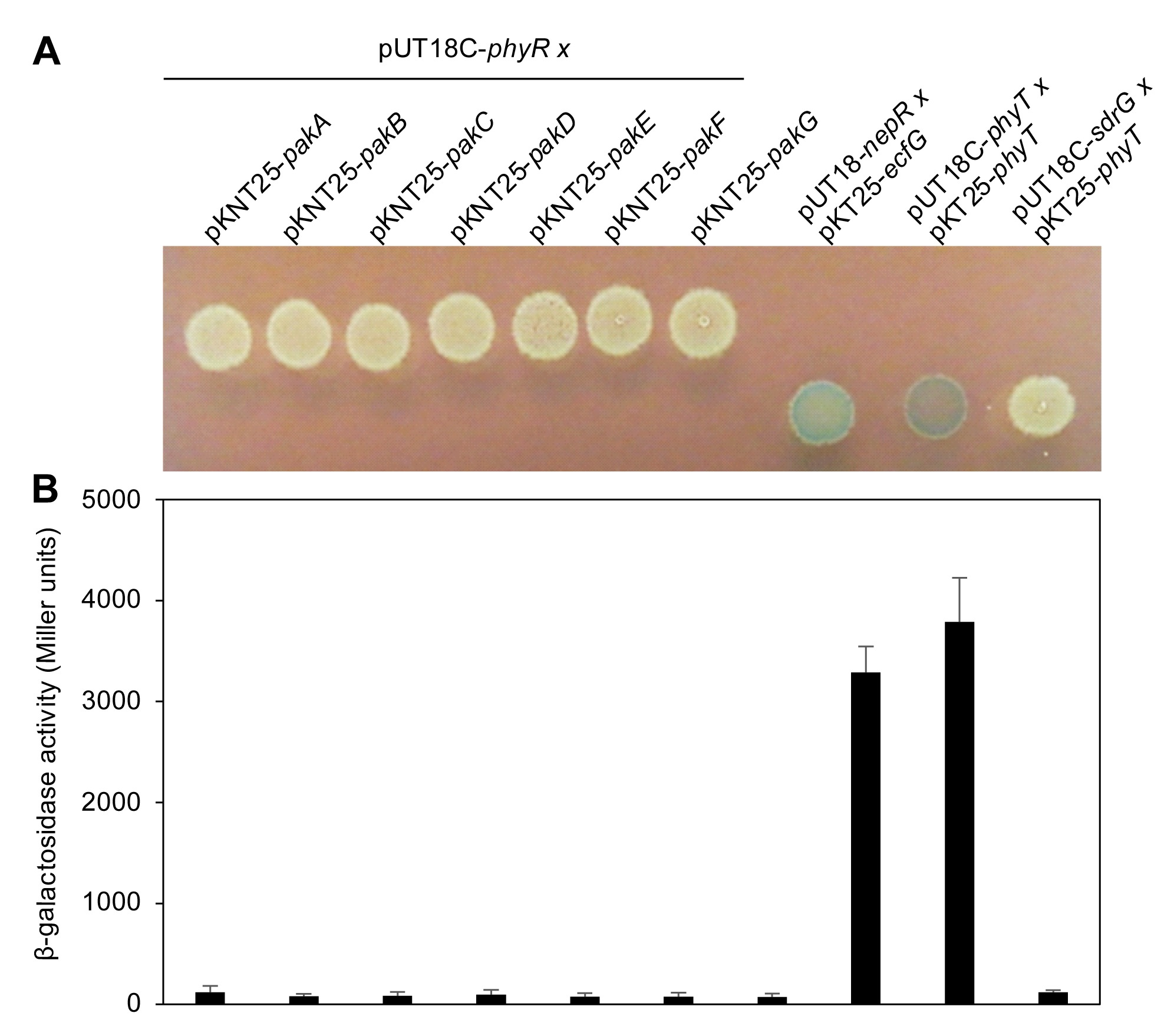

Supplement: S4 Fig — (A) BACTH assay with bacteria spotted onto LB plates containing X-Gal (40 μg/mL), IPTG (0.5 mM), and antibiotics for selection. Interactions of the C-terminal T18-PhyR wild-type with N-terminal T25-Pak fusions were tested. Stable interaction between N-terminal T18-NepR fusion and C-terminal T25-EcfG fusion was confirmed as a control. PhyT dimerization was shown with C-terminal fusion proteins. SdrG-PhyT interaction was tested with C-terminal T18-SdrG and C-terminal T25-PhyT fusion proteins. Pictures were taken after 24 h of incubation at 30°C. Blue colonies indicate protein interaction. (B) β-galactosidase assays were performed for quantification in three biological replicates. Overnight cultures containing 0.5 mM IPTG and antibiotics for selection, were inoculated from single colonies of the co-transformed bacteria and incubated at 30°C. (TIF) [file pgen.1007294.s004.tif]

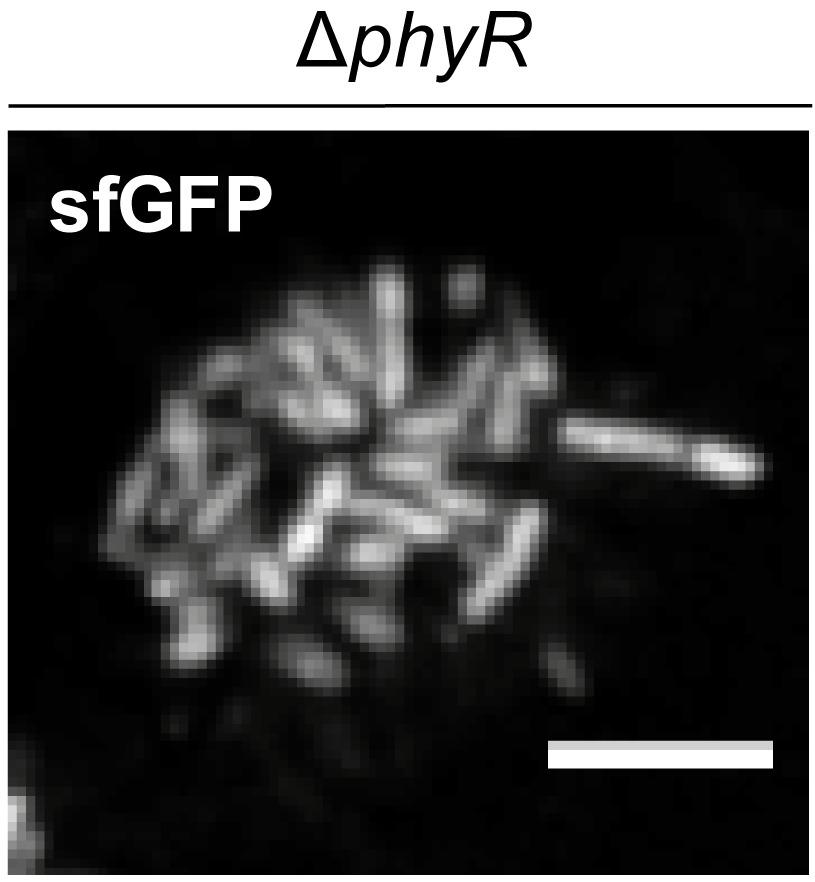

Supplement: S5 Fig — Spinning-disc confocal image of the S. melonis Fr1 ΔphyR knockout mutant upon production of sfGFP, which was induced by addition of 25 μM cumate for 12 min. Scale bar, 5 μm. (TIF) [file pgen.1007294.s005.tif]
