# Supplementary material for: Phosphorelay through the bifunctional phosphotransferase PhyT controls the general stress response in an alphaproteobacterium
Source: PLoS Genet. 2018 Apr 13;14(4):e1007294. doi: 10.1371/journal.pgen.1007294 (PMC5898713; doi:10.1371/journal.pgen.1007294)
Supplement: S1 Table — (DOCX) [file pgen.1007294.s006.docx]

**S1 Table.** Plasmids and Strains

| **Plasmids** | **Genotype and/or relevant features** | **Reference or source** |
| --- | --- | --- |
| pASK-IBA3; amp^r^ | Plasmid for tetracycline-inducible protein expression encoding a C-terminal Strep-tag, amp^r^ | IBA Lifesciences |
| pASK-IBA3-*sdrG*-*Strep*; amp^r^ | Plasmid with tetracycline-inducible SdrG-Strep production; amp^r^ | This study |
| pET28b | pET28b for IPTG-inducible protein expression; kan^r^ | EMD Biosciences, Darmstadt, Germany |
| pET28b-*sdrG*-*Strep* | IPTG-inducible SdrG-Strep production; kan^r^ | This study |
| pET26bII-*phyT-His_6_* | Derivate of pET26b for IPTG-inducible PhyT-His_6_ production with C-terminal thrombin cleavage site; kan^r^ | [14] |
| pET26bII-*phyT (H341A)-His_6_* | Derivate of pET26b for IPTG-inducible PhyT (H341A)-His_6_ production with C-terminal thrombin cleavage site; kan^r^ | [14] |
| pET26bII-*phyR-His_6_* | Derivate of pET26b for IPTG-inducible PhyR-His_6_ production with C-terminal thrombin cleavage site; kan^r^ | [14] |
| pET26bII-*nepR-His_6_* | Derivate of pET26b for IPTG-inducible NepR-His_6_ production with C-terminal thrombin cleavage site; kan^r^ | [14] |
| pDEST-*His_6_-MBP*-*pakF* | Destination plasmid for Gateway cloning with IPTG-inducible production of His_6_-MBP-tagged-PakF; carb^r^ | [19] |
| pKT25 | Plasmid for BACTH encoding *B. pertussis* CyaA T25 fragment for C-terminal fusions, kan^r^ | [42] |
| pKNT25 | Plasmid for BACTH encoding *B. pertussis* CyaA T25 fragment for N-terminal fusions, kan^r^ | [42] |
| pUT18 | Plasmid for BACTH encoding *B. pertussis* CyaA T18 fragment for N-terminal fusions, carb^r^ | [42] |
| pUT18C | Plasmid for BACTH encoding *B. pertussis* CyaA T18 fragment for C-terminal fusions, carb^r^ | [42] |
| pUT18C-*sdrG* | T18-SdrG production (C-terminal); carb^r^ | This study |
| pUT18C-*phyT* | T18-PhyT production (C-terminal); carb^r^ | This study |
| pKT25-*phyT* | T25-PhyT production (C-terminal); kan^r^ | This study |
| pKT25-*ecfG* | T25-EcfG production (C-terminal); kan^r^ | This study |
| pUT18-*nepR* | T18-NepR production (N-terminal); carb^r^ | This study |
| pKNT25-*nepR* | T25-NepR production (N-terminal); kan^r^ | Francez-Charlot (unpublished) |
| pUT18C-*phyR* | T18-PhyR production (C-terminal); carb^r^ | Francez-Charlot (unpublished) |
| pUT18C-*phyR* D194A | T18-PhyR (D194A) production (C-terminal); carb^r^ | This study |
| pUT18C-*phyR* E235A | T18-PhyR (E235A) production (C-terminal); carb^r^ | This study |
| pUT18C-*phyR* D194A, E235A | T18-PhyR (D194A;E235A) production (C-terminal); carb^r^ | This study |
| pKNT25-*pakA* | T25-PakA production (N-terminal) kan^r^ | [19] |
| pKNT25-*pakB* | T25-PakB production (N-terminal) kan^r^ | [19] |
| pKNT25-*pakC* | T25-PakC production (N-terminal) kan^r^ | [19] |
| pKNT25-*pakD* | T25-PakD production (N-terminal) kan^r^ | [19] |
| pKNT25-*pakE* | T25-PakE production (N-terminal) kan^r^ | [19] |
| pKNT25-*pakF* | T25-PakF production (N-terminal) kan^r^ | [19] |
| pKNT25-*pakG* | T25-PakG production (N-terminal) kan^r^ | [19] |
| pAK405 | Plasmid for markerless gene deletion; kan^r^ | [41] |
| pAK405-*sdrG* | Plasmid for markerless deletion of *sdrG*; kan^r^ | [19] |
| pAK405-*pakA* | Plasmid for markerless deletion of *pakA;* kan^r^ | [19] |
| pAK405-*phyT* | Plasmid for markerless deletion of *phyT*; kan^r^ | This study |
| pAK405-*phyR* | Plasmid for markerless deletion of *phyR*; kan^r^ | This study |
| pQY-*sfGFP* | Plasmid for cumate-inducible production of sfGFP; tet^r^ | This study |
| pQYD-*sfGFP-phyR* | Plasmid for cumate-inducible production of sfGFP-PhyR; tet^r^ | This study |
| pAK200-*phyT* | Plasmid for cumate-inducible production of PhyT; kan^r^ | Kaczmarczyk  (unpublished) |
| pAK200-*phyT* (H341A) | Plasmid for cumate-inducible production of PhyT (H341A); kan^r^ | This study |
| pVH-*sfGFP*-*phyR* | Plasmid for vanillate-inducible production of sfGFP-PhyR; tet^r^ | This study |
| pVH; tet^r^ | Plasmid for vanillate-inducible protein expression; tet^r^ | [44] |
| pVH-*sdrG* | Plasmid for vanillate-inducible production of HA-SdrG; tet^r^ | This study |
| pVH-*sdrG* (D56A) | Plasmid for vanillate-inducible production of HA-SdrG (D56A); tet^r^ | This study |
| pVH-*sdrG* (D56E) | Plasmid for vanillate-inducible production of HA-SdrG (D56E); tet^r^ | This study |
| pQY | Plasmid for cumate-inducible production of YFP; tet^r^ | [43] |
| pQYD-*pakA* | Plasmid for cumate-inducible production of YFP-PakA; tet^r^ | [19] |
| pQH | Plasmid for cumate-inducible protein expression; tet^r^ | [43] |
| pQH-*phyP* | Plasmid for cumate-inducible PhyP (new PhyT) production; tet^r^ | [43] |
| pTE100-*sfGFP* | Biobrick plasmid encoding *sfGFP*; tet^r^ | This study |
| pAK501-*pnhaA2-lacZ* | Reporter plasmid harboring a *nhaA2* promoter-*lacZ* transcriptional fusion; cm^r^ | [19] |
| kan^r^, kanamycin resistance; amp^r^, ampicillin resistance carb^r^, carbenicillin resistance; tet^r^ tetracycline resistance.; cm^r^ chloramphenicol resistance | | |
| ***E. coli* strains** |  |  |
| BTH101 | *F^-^, cya-99, araD139, galE15, galK16, rpsL1, hadR2, mcrA1, mcrB1* | Euromedex |
| BL21-Gold (DE3) | *E. coli B F^–^ ompT hsdS(rB^–^ mB^–^) dcm^+^ Tet^r^ gal λ(DE3) endA Hte* | Agilent |
|  |  |  |
| DH5α | *fhuA2 lac* (Δ)*U169 phoA glnV44 ϕ80' lacZ* (Δ)*M15 gyrA96 recA1 relA1 endA1 thi-1 hsdR17* | Invitrogen |
| ***S. melonis* Fr1 strains** |  |  |
| JVZ857 | *Sphingomonas melonis* Fr1 WT strain | [22] |
| JVZ 4557 | JVZ857 lacking 2 extrachromosomal plasmids; contig 3 and 4 | This study |
| JVZ4581 | JVZ4557 Δ*phyR* | This study |
| JVZ4584 | JVZ4557 Δ*sdrG* | This study |
| JVZ 2453 | JVZ857 Δ*pakC*Δ*pakB*Δ*pakE*Δ*pakF*Δ*pakG* | [19] |
| JVZ2575 | JVZ2453 Δ*sdrG* | Kaczmarczyk (unpublished) |
| JVZ4556 | JVZ2453 Δ*pakA* | This study |
| JVZ4583 | JVZ4556 Δ*sdrG* | This study |
| JVZ4582 | JVZ4556 Δ*phyT* | This study |
| JVZ3242 | JVZ2453 Δ*phyT* | [19] |
