# Supplementary material for: Phosphorelay through the bifunctional phosphotransferase PhyT controls the general stress response in an alphaproteobacterium
Source: PLoS Genet. 2018 Apr 13;14(4):e1007294. doi: 10.1371/journal.pgen.1007294 (PMC5898713; doi:10.1371/journal.pgen.1007294)
Supplement: S2 Table — (DOCX) [file pgen.1007294.s007.docx]

**S2 Table.** Primers for plasmid construction and site-directed mutagenesis

| **Primer name** | **Sequence (5' -3')** | **Description** |
| --- | --- | --- |
| **Oligonucleotides for plasmid construction** | | |
| NepR_fwd | AATTTCTAGAGATGTTGGATTTGCCCGGCAAC | BACTH |
| NepR_rev | AATTGGTACCGCGGCGAGCTTGTTCAGCAGG | BACTH |
| PhyT_fwd | AATTTCTAGAGATGGCCTCCCAACCCGACG | BACTH |
| PhyT_rev | AATTGGTACCGCATCGCGGCCCATCACGGC | BACTH |
| SdrG_fwd | AATTTCTAGAGATGTCTGCGCTCACCCAGATC | BACTH |
| SdrG_rev | AATTGGTACCGCCAGCGCGGCGAGAGCCTTG | BACTH |
| EcfG_fwd | AATTTCTAGAGATGACCCAGCCCGCTACGC | BACTH |
| EcfG_rev | AATTGGTACCGCGCGCCCCCGGCTGAGTTCG | BACTH |
| SdrG_Strep (HindIII) rev | CACAGGTCAAGCTTATTATTTTTCGAACT | sdrG-*strep* in pET28b |
| SdrG_Strep (PciI) fwd | GGCAAACATGTCTGCGCTCACCCAGA | *sdrG*-*strep* in pET28b |
| SdrG fwd in pASK‑IBA3 | GCTGGATCCATGTCTGCGCTCACC | *sdrG* in pASK‑IBA3 |
| SdrG rev in pASK‑IBA3 | GCTCCATGGAAGCTACCACGCGGTAC | SdrG in pASK‑IBA3 |
| sfGFP fwd (HindIII) | AGCCAAGCTTCCGCATGAGCAAAGG | *sfGFP* in pQY or pQYD-*phyR* |
| sfGFP rev (AseI) | AGCCATTAATTTTATACAGTTCGTCCATACC | *sfGFP* in pQY or pQYD-*phyR* |
| sfGFP-phyR fwd (HindIII) | AATTAAGCTTCCGCATGAGCAAAGGCGAGGAATTATTCAC | *sfGFP-phyR* in pVH |
| sfGFP-phyR rev (XhoI) | AATTCTCGAGTCACGCAACCGCCGTCGGCACCGTC | *sfGFP-phyR* in pVH |
| PhyR HR1 fwd (KpnI) | AAATTGGTACCGTCGATTGCTGGGCGAACAC | Knockout of *phyR* |
| PhyR HR1 rev | CCGCTACGCCGTCACGCAACAAGCGACATGGGTCGTGTTC | Knockout of *phyR* |
| PhyR HR2 fwd | GTTGCGTGACGGCGTAGCGGGCGTGG | Knockout of *phyR* |
| PhyR HR2 rev (HindIII) | AATTAAGCTTCGCTGCATGTCGCCCAGTTC | Knockout of *phyR* |
| HR1 PhyT fwd (BamHI) | AAATTGGATCCAGCCTTCCATGGCGGTTAGC | Knockout of *phyT* |
| HR1 PhyT rev | GGAGGCCATGCCCCCTGCTAGGCGAGCTTG | Knockout of *phyT* |
| HR2 PhyT fwd | CAGGGGGCATGGCCTCCTGACGGCTTATCGACGATAAAGATTC | Knockout of *phyT* |
| HR2 PhyT rev (HindIII) | AGCCAAGCTTCTTCTGCCGGTGTTGCTGATG | Knockout of *phyT* |
| **Oligonucleotides for side-directed mutagenesis** | | |
| PhyR D194A_fwd | CCCCGGTCTGGTGCTGGCGGCCATCCAGCTGGCCGACG | Mutagenesis of Asp-194 in *phyR* |
| PhyR D194A_rev | CGTCGGCCAGCTGGATGGCCGCCAGCACCAGACCGGG | Mutagenesis of Asp-194 in *phyR* |
| PhyR E235A_fwd | GCTGACCGGCGAGCGGCCCGCGCCGACGTTCCTCATCACC | Mutagenesis of Glu-235 in *phyR* |
| PhyR E235A_rev | GGTGATGAGGAACGTCGGCGCGGGCCGCTCGCCGGTCAGC | Mutagenesis of Glu-235 in *phyR* |
| SdrG D56E fwd | ATCGACGCTGCGATCCTCGAGGTCAACCTGCGCGGCGGT | Mutagenesis of Asp-56 in *sdrG* |
| SdrG D56E rev | ACCGCCGCGCAGGTTGACCTCGAGGATCGCAGCGTCGAT | Mutagenesis of Asp-56 in *sdrG* |
| SdrG D56A fwd | ATCGACGCTGCGATCCTCGCCGTCAACCTGCGCGGCGGT | Mutagenesis of Asp-56 in *sdrG* |
| SdrG D56A rev | ACCGCCGCGCAGGTTGACGGCGAGGATCGCAGCGTCGAT | Mutagenesis of Asp-56 in *sdrG* |
| PhyT H341A fwd | GCGAAGTCCATGCCCGCGTAAAGAACAACCTCCAGGTC | Mutagenesis of His-341 in p*hyT* |
| PhyT H341A rev | GACCTGGAGGTTGTTCTTTACGCGGGCATGGACTTCGC | Mutagenesis of His-341 in p*hyT* |
